# N-Alkylation of Anthracycline Antibiotics by Natural Sesquiterpene Lactones as a Way to Obtain Antitumor Agents with Reduced Side Effects

**DOI:** 10.3390/biomedicines9050547

**Published:** 2021-05-13

**Authors:** Margarita Neganova, Alexey Semakov, Yulia Aleksandrova, Ekaterina Yandulova, Sergey Pukhov, Lada Anikina, Sergey Klochkov

**Affiliations:** Institute of Physiologically Active Compounds, Russian Academy of Sciences, 142432 Chernogolovka, Russia; neganova83@mail.ru (M.N.); l_vok@list.ru (A.S.); yulia.aleks.97@mail.ru (Y.A.); yandulovacaterina@gmail.com (E.Y.); pukhov.sergey@gmail.com (S.P.); anikina1970@gmail.com (L.A.)

**Keywords:** doxorubicin, daunorubicin, sesquiterpene lactones, conjugates, cancer, cardiotoxicity, glycolysis, rat heart mitochondria

## Abstract

Anthracycline antitumor antibiotics are one of the promising classes of chemotherapeutic agents for cancer treatment. The main deterrent to their use is high toxicity to a healthy environment, including cumulative cardiotoxicity. In our work, bipharmacophore molecules containing in their structure a fragment of the known anthracycline antibiotics daunorubicin and doxorubicin and natural sesquiterpene lactones were obtained for the first time. When studying the biological activity of the synthesized compounds, it was found that with equal and, in some cases, higher cytotoxicity and glycolysis inhibition by anthracycline antibiotics conjugates with sesquiterpene lactones in comparison with doxo- and daunorubicin, a reduced damaging effect on the functioning of rat heart mitochondria was observed. The results obtained allow us to confirm the assumption that the chemical modification of the anthracycline antibiotics molecules doxo- and daunorubicin by natural sesquiterpene lactones can be a promising strategy for creating potential antitumor chemotherapeutic drugs with a pronounced cytotoxic effect on tumor cells and a reduced damaging effect on healthy cells of the human organism.

## 1. Introduction

Anthracycline antibiotics have pronounced antitumor properties. The clinical use of representatives of this class as antineoplastic agents dates to the 60s of the last century. Notable examples are doxorubicin, which is an important component of therapy aimed at the treatment of various solid tumors, soft tissue sarcomas, and aggressive lymphomas, and daunorubicin, which is active against acute lymphoblastic and myeloblastic leukemias [1]. Despite the fact that anthracyclines undoubtedly play one of the key roles in the treatment of malignant neoplasms, their use is limited due to their low selectivity of action and high toxicity in relation to healthy tissues, mainly of the cardiovascular system. Thus, chronic administration of substances of this class leads to the development of cardiomyopathy and treatment-resistant congestive heart failure, which is caused by damage to the cell myocardium membranes [2]. In this regard, for more than 40 years, scientists have been making numerous attempts to identify new anthracycline antitumor antibiotics that could surpass doxo- and daunorubicin in terms of activity and/or tolerance to the development of cardiovascular pathologies. To date, more than two thousand analogs of anthracycline antibiotics are known, which were created by various chemical modifications, substitutions, and/or conjugations, both in the tetracyclic nucleus and in the side chain or amino sugar [1], but only a few of them have become commercially successful and remain in use. Epirubicin, a semisynthetic derivative of doxorubicin obtained as a result of the hydroxyl group epimerization at the 4-position of daunosamine, and idarubicin, one of the first synthetic derivatives of daunorubicin, formed by the removal of the methoxy group in aglycone, are very popular chemotherapeutic agents, which show improvements in efficacy and cardiotoxicity [3,4,5]. This confirms the promise of the search for antitumor agents in analogs of this class of compounds.

**Figure 1:**
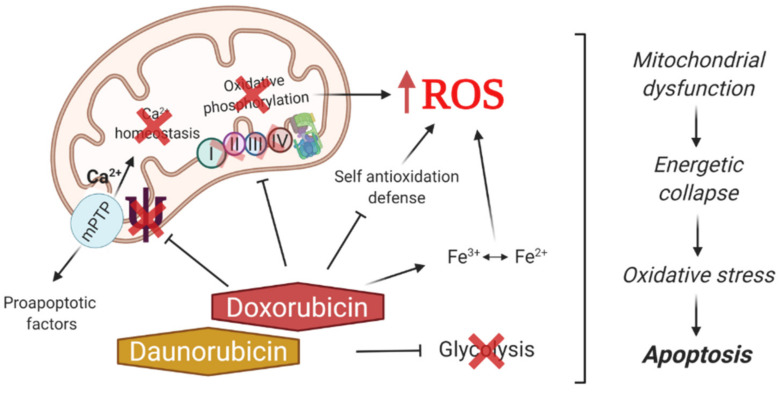
Mechanisms of antitumor action of anthracycline antibiotics associated with hyperproduction of reactive oxygen species (ROS) and inhibition of glycolysis.

It is known that the cytotoxicity of anthracycline antibiotics is caused by DNA damage, mediated mainly by the inhibition of topoisomerase II [6], as well as the excessive formation of reactive oxygen species [7] (Figure 1). Thus, one of the main causes of oxidative stress caused by this class of compounds is a violation of mitochondrial function, affecting the electron transport chain complexes of organelles, leading to their deactivation and overproduction of ROS [8,9]. Ca^2+^ homeostasis disruption triggered by anthracycline antibiotics causes the opening of the mitochondrial membrane permeability transition pore (mPTP) and a decrease in the transmembrane potential along with an increase in the permeability of the outer mitochondrial membrane for the release of apoptotic factors [10]. In addition, it has been shown that doxo- and daunorubicin are capable of inhibiting the glycolytic pathway of energy production [11], disrupting the work of the endogenous antioxidant system of cell defense [12], and also directly interacting with iron ions, forming reactive anthracycline-iron complexes, which leads to cyclic transition of Fe^3+^ and Fe^2+^, energy collapse, and additionally stimulates the ROS formation [13].

The development of anthracycline analogues is still actively continuing to reduce the risk of cardiotoxicity and improve the therapeutic index. An interesting object used for their modification can be natural origin products—sesquiterpene lactones, which contain activated double bonds in their structure and exhibit a wide range of biological activity. The antitumor potential of this class of compounds is confirmed by their ability to modulate a number of mechanisms involved in the development of oncological diseases, in particular, the processes associated with oxidative stress [14,15,16], the mitochondria functioning [17], and the glycolytic profile of transformed cells [18,19].

In this work, a number of anthracycline antibiotics conjugates with natural sesquiterpene lactones of *Inula helenium* L. (*Asteraceae*) were first obtained and studied. The anthracycline antibiotics doxorubicin (**a**) and daunorubicin (**b**) were modified with sesquiterpene lactones—isoalantholactone (**1**), alantolactone (**2**), alloalantholactone (**3**), epoxy-isoalantholactone (**4**), epoxyalantholactolactone (**5**), epoxyalloalantholactone (**6**), 6-hydroxyxanthanodiene (**7**), alanthodiene (**8**). Conjugates **1-6a-b** were obtained in our previous works [20,21,22]; compounds **7a**, **b** and **8a**, **b** were first obtained and studied. Their cytotoxic and antiproliferative properties in relation to tumor cell lines were evaluated and their influence on key aspects of formation of cytotoxicity—mitochondrial functional characteristics (mitochondrial swelling, membrane potential, and mitochondrial respiratory chain complexes) as well as the glycolytic function parameters of HeLa tumor cells— was studied.

## 2. Materials and Methods 

### 2.1. Chemistry

^1^H and ^13^C NMR spectra were recorded on Bruker AVANCE III instruments (operating frequency 500.13 and 125.78 MHz) and Bruker DPX-200 (200.13 and 50.32 MHz). In CDCl_3_, the internal standard is the residual solvent signal; subscripts “alpha” and “beta” denote nonequivalent protons at one carbon atom. To refine the multiplicity in the proton spectra analysis, we used the multiplication by the TRAF function. For some weakly intense ^13^C spectra, the convolution difference was used to increase the signal-to-noise ratio. High-resolution mass spectra were recorded using a Sciex QStar quadrupole time-of-flight mass spectrometer with orthogonal ion input and a Thermo Fisher Exactive mass spectrometer with an electrospray ionization source and Orbitrap mass analyzer. For ionization, solutions of the starting substances in acetonitrile with a concentration of ~10^−5^ M were taken. When calculating the molecular masses, the following atomic masses were used: H—1.007825, O—15.994915, C—12.000000, N—14.003074. The compounds were purified by silica gel column chromatography. If necessary, the individual components were purified by semi-preparative HPLC (Turbo LC 200 chromatograph, Perkin Elmer, Germany), diode matrix detector, UV 254 nm; analytical column 4 × 100 mm with Kromasil C18, 5 μm; preparative column 10 × 250 mm with Kromasil C18, 5 μm); gradient elution: eluent A was 0.1% trifluoroacetic acid in distilled water (pH 2.0), eluent B was acetonitrile, and the elution rates were 1 and 4 mL·min^−1^ for the analytical and preparative columns, respectively. The melting points were measured by a Kruss M3000 apparatus. TLC was performed by Alugram Xtra Sil G/UV254 plates; compounds were detected without additional treatment, as well as after treatment with an anisaldehyde reagent. The reaction of the obtained compounds was monitored using TLC on Alugram Xtra SIL D/UV254 plates in the CHCl_3_:MeOH = 10:1 system, the conversion degree of the initial lactone was controlled by ^1^H NMR (the disappearance of proton signals of the exomethylene group in the 4–5 ppm).

In chemical experiments, we used reagents from Acros Organics, Catrosa, Panreac, and Sigma–Aldrich; doxo- and daunorubicin were obtained from OSEIB (OSEIB, Kirov, Russia).

**Figure 2:**
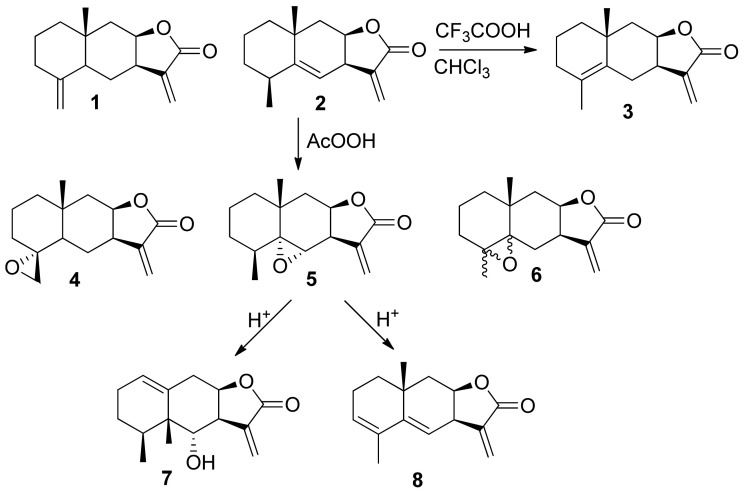
Scheme for obtaining of sesquiterpene lactones used to modify anthracycline antibiotics.

The natural sesquiterpene lactones (**1**) and (**2**) were isolated from the roots of elecampane *Inula helenium* L. according to the method we developed earlier [23]. Alloalantholactone (**3**) was obtained by isomerization of isoalantholactone (**1**) under the action of a 5-fold excess of trifluoroacetic acid (in chloroform) [22]. Epoxylactones (**4**), (**5**), (**6**) were obtained from them by the action of peracetic acid according to the procedures from [23]. 6-Hydroxyxanthanodiene (**7**) and alanthodiene (**8**) were obtained as we described earlier under the conditions of acid isomerization of epoxyalantolactone. The spectral characteristics were consistent with the literature data [23] (Figure 2).

### 2.2. Preparation of Daunorubicin and Doxorubicin Conjugates

Anthracycline antibiotics conjugates (**1a**), (**2a**), (**4a)**, (**5a**), (**6a**), (**1b**), (**3b**), (**4b**), (**5b**), (**6b**) were obtained as described earlier; the spectral characteristics corresponded to the literature data [20,21,22].

### 2.3. General Procedure for the Preparation of Doxorubicin Conjugates

In a mixture of 2 mL of methanol and 2 mL of chloroform, 0.431 mmol of the respective lactone (**1**)–(**8**) was dissolved, then 250 mg of doxorubicin hydrochloride and 120 μL (2 eq.) of triethylamine were added and left at room temperature for seven days. The reaction was monitored by TLC in the CHCl_3_:MeOH = 10:1 system. The reaction mixture was evaporated under reduced pressure on a rotary evaporator, purified by column chromatography on silica gel, washed first with CHCl_3_, then CHCl_3_:AcOEt = 10:2 and CHCl_3_:MeOH = 10:1, and the product was eluted from the column with a narrow zone. If necessary, additional HPLC purification was carried out.

### 2.4. Preparation of Daunorubicin as a Base

In a separatory funnel, 1 g of daunorubicin hydrochloride was dissolved in 200 mL of water; 100 mL of chloroform and 1–5 mL of methanol were added, and NaHCO_3_ was added until the main part of the colored anthracycline was transferred to the organic layer. The lower layer was poured off and evaporated, and the remaining daunorubicin was extracted from the aqueous phase three times with 50 mL of chloroform. The sum of the chloroform extracts was evaporated to yield daunorubicin base powder.

### 2.5. General Procedure for the Preparation of Daunorubicin Conjugates

In a mixture of 2 mL of methanol and 2 mL of chloroform, 0.43 mmol of the corresponding lactone (1)–(8) was dissolved, and hen 250 mg of daunorubicin was added in the form of a free amine and left at room temperature for seven days. The progress of the reaction was monitored by TLC in the CHCl_3_:MeOH = 10:1 system. The reaction mixture was evaporated under reduced pressure on a rotary evaporator, purified by column chromatography on silica gel, washed first with CHCl_3_ (until a low-polarity colored aglycone daunorubicin emerged), then with CHCl_3_:AcOEt = 10:2 and CHCl_3_:MeOH = 10:1, and the colored product was eluted from the column in a narrow zone (unreacted daunorubicin remained at the start). Fractions with the target product were evaporated from the solvent, and the product was evaporated once again together with ether. If necessary, additional HPLC purification was carried out.

Characterization of compounds **7a**, **7b**, **8a**, **8b** (see Supplementary Materials).

*(8S,10S)-6,8,11-Trihydroxy-10-(((2R,4S,5S,6S)-5-hydroxy-4-((((3S,3aS,4S,4aR,5S,9aR)-4-hydroxy-4a,5-dimethyl-2-oxo-2,3,3a,4,4a,5,6,7,9,9a-decahydronaphtho[2,3-b]furan-3-yl)methyl)amino)-6-methyltetrahydro-2H-pyran-2-yl)oxy)-8-(2-hydroxyacetyl)-1-methoxy-7,8,9,10-tetrahydrotetracene-5,12-dione* (**7a**). A red solid (127 mg, 37.2%): m.p. = 170–175 °C; TLC Rf = 0.39 (CHCl_3_:MeOH = 10:1); HRMS (ESI): m/z = 792.3252 (M+H^+^). C_42_H_49_NO_14_. Calculated, m/z = [M+H]^+^ = 792.3226. ^1^H-NMR (500 MHz, CDCl_3_, *δ, ppm*) 0.86 (3H, d, *J* = 6.8 Hz, H-42), 0.99 (3H, s, H-41), 1.29–1.40 (2H, m, H-36a, H-36b), 1.36 (3H, d, *J* = 6.6 Hz, H-27), 1.69–1.87 (2H, m, H-23a, H-23b), 1.93 (1H, m, H-37), 1.93–2.00 (2H, m, H-35a, H-35b), 2.14–2.19 (m, H-12a), 2.16 (dd, *J* = 15.6, 4.2 Hz, H-32a), 2.22–2.33 (1H, m, H-32b), 2.37 (1H, br.d, *J* = 14.6 Hz, H-12b), 2.55 (1H, m, H-29), 2.62 (1H, d, *J* = 7.0 Hz, H-28a), 2.81–2.96 (3H, m, H-28b, H-24, H-30), 3.03 (1H, dd, *J* = 18.6, 3.1 Hz, H-10a), 3.26 (1H, d, *J* = 18.8 Hz, H-10b), 3.65 (1H, br.s, H-25), 3.84 (1H, d, *J* = 5.0 Hz, H-39), 4.00–4.12 (1H, m, H-26), 4.08 (3H, s, H-21), 4.60 (1H, dt, *J* = 11.0, 7.5 Hz, H-31), 4.76 (2H, s, H-20), 5.32 (1H, br.s, H-13), 5.52 (1H, d, *J* = 3.1 Hz, H-22), 5.62 (1H, br.s, H-34), 7.39 (1H, d, *J* = 8.2 Hz, H-2), 7.78 (1H, dd, *J* = 7.5, 8.2 Hz, H-3), 8.02 (1H, d, *J* = 7.5 Hz, H-4), 13.24 (1H, br.s, hydroquinone OH), 13.95 (1H, br.s, hydroquinone OH); ^13^C-NMR (126 MHz, CDCl_3_, *δ, ppm*) 16.4 (C-42), 17.3 (C-27), 19.4 (C-41), 25.7 (C-35), 26.9 (C-36), 30.3 (C-23), 32.7 (C-37), 34.1 (C-10), 35.3 (C-12), 35.6 (C-32), 42.0 (C-38), 42.9 (C-30), 45.8 (C-28), 52.8 (C-24), 56.8 (C-21), 65.6 (C-20), 66.0 (C-11), 67.2 (C-25, C-26), 69.6 (C-13), 74.4 (H-39), 77.4 (C-31), 101.0 (C-22), 111.6 (C-7), 111.8 (C-16), 118.6 (C-2), 120.0 (C-4), 121.0 (C-18), 128.1 (C-34), 135.0 (C-33), 135.6 (C-5), 135.9 (C-3), 155.8 (C-8), 156.3 (C-15), 161.2 (C-1), 178.0 (C-40), 186.9 (C-6), 187.2 (C-17), 213.8 (C-19).

*(8S,10S)-10-(((2R,4S,5S,6S)-4-((((3S,3aR,8aR,9aR)-5,8a-Dimethyl-2-oxo-2,3,3a,7,8,8a,9,9a-octahydronaphtho[2,3-b]furan-3-yl)methyl)amino)-5-hydroxy-6-methyltetrahydro-2H-pyran-2-yl)oxy)-6,8,11-trihydroxy-8-(2-hydroxyacetyl)-1-methoxy-7,8,9,10-tetrahydrotetracene-5,12-dione* (**8a**). A red solid (77 mg, 23.1%): m.p. = 166–170 °C; TLC Rf = 0.54 (CHCl_3_:MeOH = 10:1); HRMS (ESI): m/z = 774.3148 (M+H^+^). C_42_H_47_NO_13_. Calculated, m/z = [M+H]^+^ = 774.3120. ^1^H-NMR (500 MHz, CDCl_3_, *δ, ppm*) 1.03 (3H, s, H-41),1.32 (1H, m, H-34a), 1.37 (3H, d, *J* = 6.6 Hz, H-27), 1.50 (1H, dd, *J* = 9.1, 2.4, Hz, H-32a), 1.51 (1H, d, *J* = 18.4 Hz, H-34b), 1.71 (3H, br.s, H-42), 1.76 (2H, m, H-23a, H-23b), 2.01 (1H, dt, *J* = 18.8, 5.6 Hz, H-35a), 2.16 (1H, dd, *J* = 13.1, 3.8 Hz, H-12a), 2.18 (1H, dd, *J* = 14.8, 3.2 Hz, H-32b), 2.25 (1H, br.d, *J* = 15.7 Hz, H-35b), 2.37 (1H, dt, *J* = 14.4, 2.2 Hz, H12b), 2.82 (1H, dd, *J* = 11.0, 5.9 Hz, H-28a), 2.87 (1H, ddd, *J* = 11.7, 5.7, 2.6 Hz, H-24), 2.95 (1H, m, H-29), 2.98 (1H, m, H-28b), 3.00 (1H, d, *J* = 18.9 Hz, H-10a), 3.24 (1H, m, H-30), 3.25 (1H, dd, *J* = 18.6, 1.8 Hz, H-10b), 3.65 (1H, br.s, H-25), 4.0–4.09 (1H, m, H-26), 4.07 (3H, s, H-21), 4.75 (2H, s, H-20), 4.77 (1H, m, H-31), 5.21 (1H, d, *J* = 3.5 Hz, H-39), 5.31 (1H, dd, *J* = 3.7, 2.2 Hz, H-13), 5.52 (1H, br.d, *J* = 2.6 Hz, H-22), 5.58 (1H, br.d, *J* = 3.4 Hz, H-36), 7.39 (1H, dd, *J* = 8.6, 1.1 Hz, H-2), 7.77 (1H, dd, *J* = 7.8, 8.4 Hz, H-3), 8.01 (1H, dd, *J* = 7.8, 1.0 Hz, H-4), 13.22 (1H, br.s, hydroquinone OH), 13.24 (1H, br.s, hydroquinone OH); ^13^C-NMR (126 MHz, CDCl_3_, *δ, ppm*) 17.3 (C-27), 20.3 (C-42), 22.3 (C-35), 24.8 (C-41), 30.6 (C-33), 30.9 (C-23), 34.1 (C-10), 35.6 (C-12), 37.3 (C-34), 38.3 (C-30), 39.6 (C-32), 42.6 (C-28), 45.8 (C-29), 52.4 (C-24), 56.8 (C-21), 65.6 (C-20), 67.2 (C-26), 67.4 (C-25), 69.7 (C-13), 76.8 (C-11), 77.4 (C-31), 101.2 (C-22), 111.6 (C-7), 111.7 (C-16), 113.0 (C-39), 118.6 (C-2), 120.0 (C-4), 121.0 (C-18), 126.9 (C-36), 130.4 (C-37), 133.6 (C-9), 134.0 (C-14), 135.6 (C-5), 135.9 (C-3), 145.0 (C-38), 155.8 (C-8), 156.3 (C-15), 161.2 (C-1), 177.8 (C-40), 186.9 (C-6), 187.2 (C-17), 213.8 (C-19).

*(8S,10S)-8-Acetyl-6,8,11-trihydroxy-10-(((2R,4S,5S,6S)-5-hydroxy-4-((((3S,3aS,4S,4aR,5S,9aR)-4-hydroxy-4a,5-dimethyl-2-oxo-2,3,3a,4,4a,5,6,7,9,9a-decahydronaphtho[2,3-b]furan-3-yl)methyl)amino)-6-methyltetrahydro-2H-pyran-2-yl)oxy)-1-methoxy-7,8,9,10-tetrahydrotetracene-5,12-dione* (**7b**). A red solid (183 mg, 54.7%): m.p. = 155–162 °C; TLC Rf = 0.78 (CHCl_3_:MeOH = 10:1); HRMS (ESI): m/z = 776.3291 (M+H^+^). C_42_H_49_NO_13_. Calculated, m/z = [M+H]^+^ = 776.3277. ^1^H-NMR (500 MHz, CDCl_3_, *δ, ppm*) 0.86 (3H, d, *J* = 6.8 Hz, H-42), 0.98 (3H, s, H-41), 1.36 (3H, d, *J* = 6.6 Hz, H-27), 1.29–1.42 (2H, m, H-36a, H-36b), 1.69–1.77 (2H, m, H-23a, H-23b), 1.90–1.99 (2H, m, H-35a, H-35b), 1.93 (1H, m, H-37), 2.06–2.12 (1H, m, H-12a), 2.10 (1H, dd, *J* = 14.8, 4.1 Hz, H-32a), 2.23–2.32 (1H, m, H-32b), 2.35 (1H, br.d, *J* = 15.0 Hz, H-12b), 2.42 (3H, s, 3H, H-20), 2.53 (1H, m, H-29), 2.66 (1H, m, H-28a), 2.86 (1H, m, H-30), 2.91 (1H, m, H-28b), 2.96 (1H, d, *J* = 18.6 Hz, H-10a), 3.22 (1H, dt, *J* = 18.7, 2.4 Hz, H-10b), 3.65 (1H, br.d, *J* = 6.8 Hz, H-25), 3.83 (1H, d, *J* = 6.8 Hz, H-39), 4.03–4.10 (1H, m, H-26), 4.08 (3H, s, H-21), 4.60 (1H, dt, *J* = 10.8, 7.3 Hz, H-31), 5.29 (1H, dd, *J* = 3.9, 2.1 Hz, H-13), 5.50 (1H, br.d, *J* = 3.5 Hz, H-22), 5.62 (1H, dt, *J* = 3.9, 1.8 Hz, H-34), 7.38 (1H, dd, *J* = 8.6, 0.9 Hz, H-2), 7.78 (1H, dd, *J* = 7.8, 8.4 H-3), 8.02 (1H, dd, *J* = 7.7, 1.1 Hz, H-4), 13.27 (1H, br.s, hydroquinone OH), 13.96 (1H, br.s, hydroquinone OH); ^13^C-NMR (126 MHz, CDCl_3_, *δ, ppm*) 16.4 (C-42), 17.2 (C-27), 19.4 (C-41), 24.9 (C-20), 25.6 (C-35), 26.9 (C-36), 30.4 (C-23), 32.7 (C-32), 33.5 (C-10), 35.0 (C-37), 35.3 (C-12), 42.0 (C-30), 42.9 (C-38), 45.7 (C-28), 45.8 (C-29), 52.8 (C-24), 56.8 (C-21), 67.1 (C-26), 67.3 (C-25), 69.8 (C-13), 74.4 (C-39), 77.0 (C-31), 77.1 (C-11), 100.9 (C-22), 111.4 (C-7), 111.6 (C-16), 118.5 (C-2), 119.9 (C-4), 121.1 (C-18), 128.0 (C-34), 134.4 (C-9), 134.5 (C-14), 135.0 (C-33), 135.7 (C-5), 135.9 (C-3), 156.0 (C-8), 156.5 (C-15), 161.2 (C-1), 178.0 (C-40), 186.9 (C-6), 187.2 (C-17), 211.9 (C-19).

*(8S,10S)-8-Acetyl-10-(((2R,4S,5S,6S)-4-((((3S,3aR,8aR,9aR)-5,8a-dimethyl-2-oxo-2,3,3a,7,8,8a,9,9a-octahydronaphtho[2,3-b]furan-3-yl)methyl)amino)-5-hydroxy-6-methyltetrahydro-2H-pyran-2-yl)oxy)-6,8,11-trihydroxy-1-methoxy-7,8,9,10-tetrahydrotetracene-5,12-dione* (**8b**). A red solid (191 mg, 57.2%): m.p. = 152–158 °C; TLC Rf = 0.81 (CHCl_3_:MeOH = 10:1); HRMS (ESI): m/z = 758.3197 (M+H^+^). C_42_H_47_NO_12_. Calculated, m/z = [M+H]^+^ = 758.3171. ^1^H-NMR (500 MHz, CDCl_3_, *δ, ppm*) 1.03 (3H, s, H-41), 1.36 (3H, d, *J* = 6.6 Hz, H-27), 1.32 (1H, m, H-34a), 1.50 (2H, d, *J* = 18.3 Hz, H-34b + br.d, *J* = 11.8 Hz, H-32a), 1.71 (3H, br.s, H-42), 1.75 (2H, m, H-23a, H-23b), 2.00 (1H, dt, *J* = 18.7, 5.2 Hz, H-35a), 2.10 (1H, dd, *J* = 14.8, 4.1 Hz, H-12a), 2.18 (1H, dd, *J* = 14.6, 3.1 Hz, H-32b), 2.25 (1H, br.d, *J* = 15.7 Hz, H-35b), 2.35 (1H, dt, *J* = 14.9, 2.7 Hz, H12b), 2.41 (3H, s, H-20), 2.82 (1H, m, H-28a), 2.89 (1H, m, H-24), 2.94 (1H, m, H-29), 2.93(1H, d, *J* = 19.0 Hz, H-10a), 2.97 (1H, m, H-28b), 3.20 (1H, dd, *J* = 18.8, 2.0 Hz, H-10b), 3.24 (1H, m, H-30), 3.64 (1H, d, *J* = 1.5 Hz, H-25), 4.05–4.10 (1H, m) H-26, 4.07 (3H, br.s, H-21), 4.75 (1H, dt, *J* = 5.6, 2.7 Hz, H-31), 5.20 (1H, d, *J* = 3.2 Hz, H-39), 5.28 (1H, dd, *J* = 3.8, 2.0 Hz, H-13), 5.52 (1H, t, *J* = 2.5 Hz, H-22), 5.57 (1H, d, *J* = 4.0 Hz, H-36), 7.38 (1H, dd, *J* = 8.5, 0.8 Hz, H-2), 7.77 (1H, dd, *J* = 8.4, 7.8 Hz, H-3), 8.01 (1H, dd, *J* = 7.7, 0.8 Hz, H-4), 13.26 (1H, br.s, hydroquinone OH), 13.94 (1H, br.s, hydroquinone OH); ^13^C-NMR (126 MHz, CDCl_3_, *δ, ppm*) 17.2 (C-27), 20.3 (C-42), 22.3 (C-35), 24.8 (C-41), 24.9 (C-20), 30.5 (C-33), 30.9 (C-23), 33.5 (C-10), 35.0 (C-12), 37.3 (C-34), 38.4 (C-30), 39.6 (C-32), 42.5 (C-28), 45.9 (C-29), 52.4 (C-24), 56.8 (C-21), 67.0 (C-26), 67.5 (C-25), 70.0 (C-13), 77.0 (C-11), 77.3 (C-31), 101.2 (C-22), 111.4 (C-7), 111.6 (C-16), 113.0 (C-39), 118.5 (C-2), 119.9 (C-4), 121.1 (C-18), 126.8 (C-36), 130.5 (C-37), 134.4 (C-9), 134.5 (C-14), 135.6 (C-5), 135.8 (C-3), 144.9 (C-38), 156.0 (C-8), 156.5 (C-15), 161.2 (C-1), 177.8 (C-40), 186.8 (C-6), 187.1 (C-17), 211.9 (C-19).

### 2.6. Cell Lines and Cultivation

Human cell cultures of tumor origin (A549 (lung carcinoma), HCT116 (colon adenocarcinoma), MCF7 (breast cancer), RD (embryonic rhabdomyosarcoma)), and the line of non-transformed cells HEK293 (embryonic kidney) provided by the Laboratory of Tumor Cell Genetics at the N.N. Blokhin Russian Cancer Research Institute, as well as by the Institute of Cytology of the Russian Academy of Sciences, were grown in DMEM (Gibco, Scotland, UK) and MEM (Gibco, Scotland, UK) containing fetal calf serum (10% by volume) (ThermoFisher Scientific, Paisley, UK), L-glutamine (2 mM) (Gibco, Scotland, UK), and penicillin-streptomycin (1% by volume) (PanEco, Moscow, Russia). The cultivation was carried out at 37 °C in a humidified CO_2_ atmosphere (5%).

### 2.7. Determination of Cell Viability

Cellular viability was determined by the MTT test [24]. The cells were seeded in a 96-well plate in the amount of 1 × 10^4^ cells/200 μL of complete culture medium and cultured at 37 °C in a CO_2_ atmosphere (5%). After 24 h of incubation, various concentrations of the test compounds dissolved in DMSO (≤1%) were added to the cell cultures, the solvent was added to the control wells in an equal volume, and then the cells were cultured under the same conditions for 72 h. After the incubation time, MTT (3-(4,5-dimethylthiazol-2-yl)-2,5-diphenyltetrazolium bromide, 5 mg/mL) was added to each well, and the plates were further incubated for 2 h (until a characteristic color appeared). Then, the formazan granules were dissolved in DMSO.

Using a plate analyzer (Cytation3, BioTech Instruments Inc., USA), the optical density was determined at λ = 530 nm. The concentration value causing 50% inhibition of cell population growth (IC_50_) was determined from dose-response curves using GraphPad Prism 9.0 software.

### 2.8. Animals

The experiments used male nonlinear outbred rats weighing 200–220 g. The animals were kept in a standard vivarium with a 12-hour light regime and free access to water and food. All manipulations with animals were carried out in accordance with the decisions of the IPAC RAS Bioethics Commission.

### 2.9. Rat Brain Homogenate and Heart Mitochondria

To obtain a brain homogenate, decapitation of rats anesthetized in advance with CO_2_ was performed using a guillotine. The brain was homogenized in a buffer containing KCl (120 mM) and HEPES (20 mM) (Gibco, Scotland, UK), pH = 7.4, at 4 °C and centrifuged at 1500 rpm to obtain a supernatant.

Rat heart mitochondria were isolated by differential centrifugation using ice-cold buffer containing KCl (120 mM) and HEPES (20 mM), pH = 7.4, and a trypsin inhibitor. The mitochondrial yield ranged from 5 to 10 mg/mL of protein in the heart sediment. Mitochondria were stored in plastic containers at 4 °C.

The quantitative determination of protein was carried out according to the standard technique using the microbiuret method [25].

### 2.10. Lipid Peroxidation

To study the effect of compounds on the process of lipid peroxidation (LPO) of rat brain homogenate, we used a modified version of the TBA test [26], which is based on the reaction of 2-thiobarbituric acid with the final LPO product—malonic dialdehyde (MDA). According to the experimental scheme, the well of the plate contained the studied compounds (100 μM), rat brain homogenate (2 mg/mL), as well as 500 μM ferrous iron ions (FeSO_4_ × 10H_2_O). After 30 min of incubation at 37 °C, TBA reagent was added to all samples, incubated for 90 min at 90 °C, and centrifuged at 6000 rpm for 15 min. The optical density of the selected supernatant was measured on a Victor 3 plate analyzer (Perkin Elmer, Germany city, abbreviated state (for USA/Canada), country) at λ = 540 nm.

### 2.11. Mitochondrial Swelling

Mitochondrial swelling was recorded by the light transmission of the rat heart mitochondrial suspension at λ = 540 nm using a Victor 3 plate analyzer (Perkin Elmer, Germany). The swelling rate was estimated as △A540/min and was calculated as the tangent of the angle of the steepest dependence curve part △A540 versus time.

### 2.12. Mitochondrial Membrane Potential

The transmembrane potential of rat heart mitochondria was measured by a Victor 3 plate analyzer (Perkin Elmer, Germany) using a potential-dependent indicator Safranin A [27]. The mitochondrial preparation was diluted in a buffer containing mannitol (225 mM) (Dia-M, Moscow, Russia), sucrose (75 mM) (Sigma Aldrich, Saint Louis, USA), HEPES (10 mM) (Gibco, Scotland, UK), EGTA (20 μM) (Dia-M, Moscow, Russia), KH_2_PO_4_ (1 mM), pH = 7.4, at the rate of 0.5 mg of protein in 1 mL of medium. Safranin A (5 μM) was added to the suspension immediately before the start of the measurement. The organelles were energized with a solution of potassium succinate and rotenone, against which the studied compounds were added at a concentration of 100 μM. The mitochondrial pore was induced by the addition of CaCl_2_ (25 μM).

### 2.13. The Work of Mitochondrial Respiratory Chain Complexes

The effect of the compounds on the functioning of the electron transport chain complexes was carried out using an Agilent Seahorse XF96e Analyzer (Seahorse Bioscience, Billerica, MA, USA) according to their ability to change the rate of oxygen uptake by isolated rat heart mitochondria (10 μg/well). The following modulators were used: activators of complex I of the electron transport chain, NADH dehydrogenase,—glutamate/malate; rotenone (2 μM) (Sigma Aldrich, Saint Louis, USA)—a tissue respiration blocker that stops the transfer of electrons from the reduced form of NADH to cytochrome b, was used as complex I inhibitor; potassium succinate (2 μM) (Dia-M, Moscow, Russia), which is a substrate of complex II of the electron transport chain, made it possible to assess mitochondrial respiration mediated by succinate dehydrogenase; complex III (ubiquinol-cytochrome c-oxidoreductase) inhibitor—a blocker of cellular respiration antimycin A (Sigma Aldrich, Saint Louis, USA), as well as ascorbate/TMPD (0.5 μM) (Sigma Aldrich, Saint Louis, USA), allowed us to evaluate the cytochrome-c-oxidase mediated respiration of organelles.

### 2.14. Parameters of the Glycolytic Function of the Tumor Cells HeLa

The ability of the studied compounds to modulate anaerobic glycolysis was investigated using the Agilent Seahorse XF96e Analyzer (Seahorse Bioscience, Billerica, MA, USA) according to the level of hydrogen proton production in the samples on the HeLa cervical tumor cell line by the glycolysis stress test [28].

HeLa cells in the exponential growth phase were plated into a 96-well cell culture microplate Seahorse (the density of the cell culture was 3 × 10^4^/well). After 24 h, the rate of medium extracellular acidification by cells treated with the test compounds was recorded in real time during the sequential injection of modulators: glucose (10 mM) (Dia-M, Moscow, Russia), oligomycin (1 μM) (Sigma Aldrich, Saint Louis, USA), 2-fluoro-2-deoxy-d-glucose (25 mM) (Sigma Aldrich, Saint Louis, USA). This allowed us to estimate the glycolysis intensity in cells by three main parameters of glycolytic function: glycolysis by adding saturating amounts of glucose to the system, glycolytic capacity by the introduction of oligomycin, and glycolytic reserve by using the inhibitor of this glycolysis 2-fluoro-2-deoxy-d-glucose.

## 3. Results & Discussion

### 3.1. Chemical Synthesis

**Figure 3:**
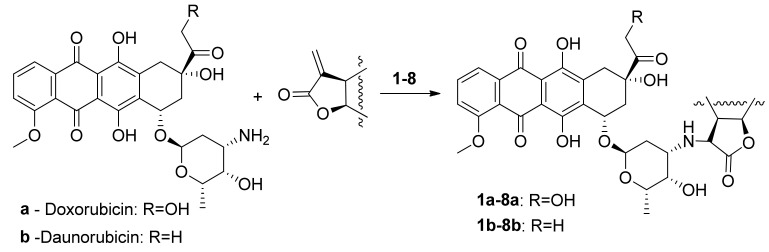
Scheme for the preparation of conjugates of daunorubicin and doxorubicin with sesquiterpene lactones.

All used lactones contain an exocyclic electron-deficient double bond in the lactone cycle and can enter into the aza-Michael reaction with amines. Anthracycline antibiotics containing an amino group in the carbohydrate fragment, doxorubicin (**a**), and daunorubicin (**b**) were used as such amines. Before carrying out the aza-Michael reaction, daunorubicin hydrochloride was converted to a base. The resulting amine is more reactive and is easily isolated in pure form by extraction with chloroform from an aqueous solution. Doxorubicin is more hydrophilic, and its base is not extracted from the aqueous solution by chloroform. In this case, to carry out the aza-Michael reaction with sesquiterpene lactones, doxorubicin was used in the form of hydrochloride, and the process was carried out in the presence of a base (triethylamine), under the action of which, in the reaction medium, doxorubicin was released in situ as a free amine and directly reacted with the lactone (Figure 3).

**Figure 4:**
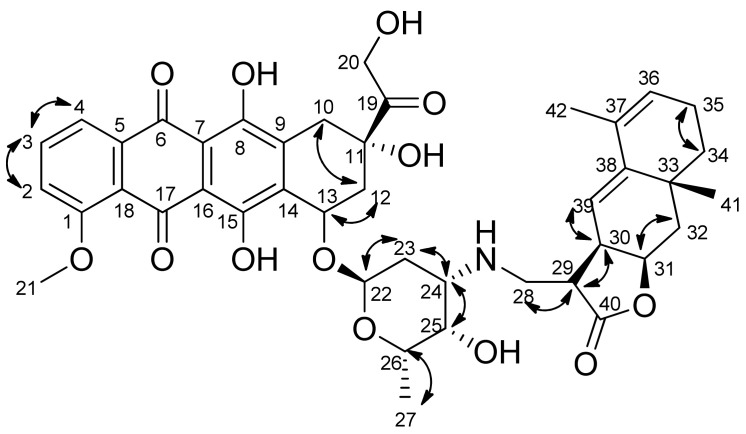
Main correlations H-H-COZY and numbering of carbons, for example, compound **8a**.

Sesquiterpene lactones (**1**)–(**8**) react both with daunorubicin and doxorubicin under mild conditions, but at a low rate. As a result, a number of previously undescribed conjugates containing two pharmacophore fragments, natural sesquiterpene lactone and anthracycline antibiotics daunorubicin or doxorubicin, were obtained. The degree of conversion of the starting lactones was monitored by TLC and ^1^H NMR (disappearance of signals from the protons of the exomethylene group—doublets at 5.4 and 6.16 ppm). The structure of the synthesized compounds was established using a set of physicochemical methods: NMR spectroscopy (including two-dimensional COZY and NOESY experiments) and high-resolution mass spectrometry. The ^1^H NMR spectra contains signals of protons appearing in the products at the C-29 atom in the range of 2.5–2.8 ppm in the form of a quartet with a spin-spin coupling (SSC) constant of 6.6–6.9 Hz. The signals and their multiplicity of the remaining protons of both the initial lactone and daunorubicin or doxorubicin are retained, which corresponds to the proposed structures, as well as the configuration of all asymmetric centers of the initial molecules. In addition, a new chiral center with a certain configuration is formed in the conjugates [20]. The stereoconfiguration of the new asymmetric center (atom C-29) was established based on two-dimensional experiments H—H COZY and NOESY (Figure 4).

### 3.2. The Study of Biological Activity

Antitumor drugs that are planned to be used as chemotherapy for the treatment of neoplasms should have a number of specific properties, one of which is the cytotoxicity presence towards tumor cells. Therefore, at the first stage of the biological activity study of the doxorubicin and daunorubicin synthesized conjugates with sesquiterpene lactones, the cytotoxic profile was analyzed in cell cultures, including cell lines of tumor origin and the line of non-transformed cells in order to assess the compound selectivity in relation to malignant cells.

According to the obtained data, presented in Table 1, in relation to all lines of tumor cells, pronounced cytotoxic properties were found, comparable to the action of doxo- and daunorubicin, without increasing the toxic effect on normal cells for most of the conjugates. Moreover, for a number of tested compounds, an increased viability of normal human renal epithelium cells was recorded. Thus, the IC_50_ for HEK293 for doxorubicin (**a**) was 6.78 ± 0.76 μM, in turn, for its derivatives containing isoalantholactone (**1a**), alantolactone (**2a**), and alanthodiene (**8a**), values of IC_50_ more than double that of the initial cytostatic agent were shown: 19.82 ± 0.42 μM, 29.83 ± 0.18 μM, and 16.42 ± 1.07 μM, respectively. A similar situation was found for the daunorubicin conjugate with epoxyalantolactone (**5b**). The IC_50_ (**b)** value of 12.31 ± 1.17 μM, (**5b)** shows a value exceeding 18 μM.

It is also important to note that lactones themselves do not have a pronounced cytotoxic effect on tumor cells, while for modified doxorubicin derivatives, for example, (**6a**), the IC_50_ value of cytotoxicity was 0.25 ± 0.04 μM, and for daunorubicin conjugates (**4b**) the IC_50_ value was 0.02 ± 0.001 μM. This is an order of magnitude higher than the activity of the original pharmacophores. Thus, the combination of an anthracycline antibiotic with sesquiterpene lactone in one molecule makes it possible to obtain a cytotoxic effect comparable to the initial anthracyclines in relation to tumor origin cells and a reduced damaging effect on a healthy cell culture (HEK293). To understand this effect, we studied the influence of the synthesized compounds on oxidative stress (accumulation of free radicals), mitochondrial function, and glycolysis as the main metabolic pathway for obtaining energy by transformed tumor cells.

**Figure 5:**
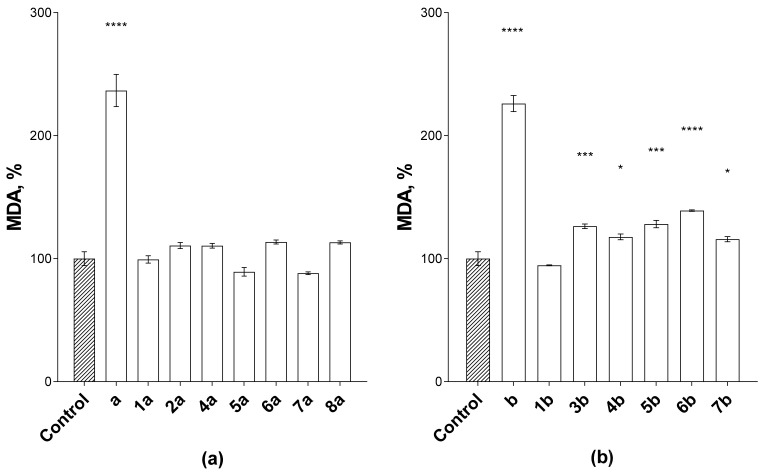
Effect of doxorubicin (**a**) and daunorubicin (**b**) conjugates with sesquiterpene lactones on the lipid peroxidation process of rat brain homogenate (2 mg/mL) initiated by ferrous iron ions (500 μM). The test compound concentration was 100 μM. Data are presented as % of MDA relative to control (DMSO ≤ 1%): as mean ± SEM (three repeated measurements for each compound). Asterisks (*, ***, ****) indicate a statistically significant (*p* < 0.05, *p* < 0.001, *p* < 0.0001) difference between the control and the studied compounds (one-way ANOVA, Dunnett’s test).

Oxidative stress and, in particular, the process of lipid peroxidation are among the main causes for cellular defect accumulation as a result of the overproduction of free radicals [29]. This can lead to tumor cell transformation and uncontrolled proliferation [30,31]. The study of the effect of the synthesized compounds on the lipid peroxidation process of the rat brain homogenate used as a model system was carried out by the TBA test. Ions of bivalent iron were used as an initiator to accelerate the reaction time for the formation of MDA. They are able to trigger the Fenton reaction with hydroxyl radical formation. A high prooxidant effect was observed for anthracycline antibiotics—doxo- and daunorubicin (the percentage of MDA was 236.68 ± 13.06 and 226.05 ± 6.60, respectively). In turn, an increase in the MDA level was not observed or did not exceed 139.08 ± 0.64% for the synthesized derivatives (Figure 5).

Mitochondria are the main free radical sources in the cell. Defects in the functioning of these organelles are manifested in electron transport chain disruption, the induction of the permeability transition pore opening, and depolarization of the mitochondrial membrane. For example, a pathological change in the activity of even one of the mitochondria electron-transport chain complexes can lead to overproduction of superoxide radicals and other reactive oxygen species. In turn, this contributes to the development of oxidative stress and permanent damage to cell components [32]. Heart mitochondria were chosen as the study object since cumulative cardiotoxicity is the main side effect of anthracycline antibiotics.

**Figure 6:**
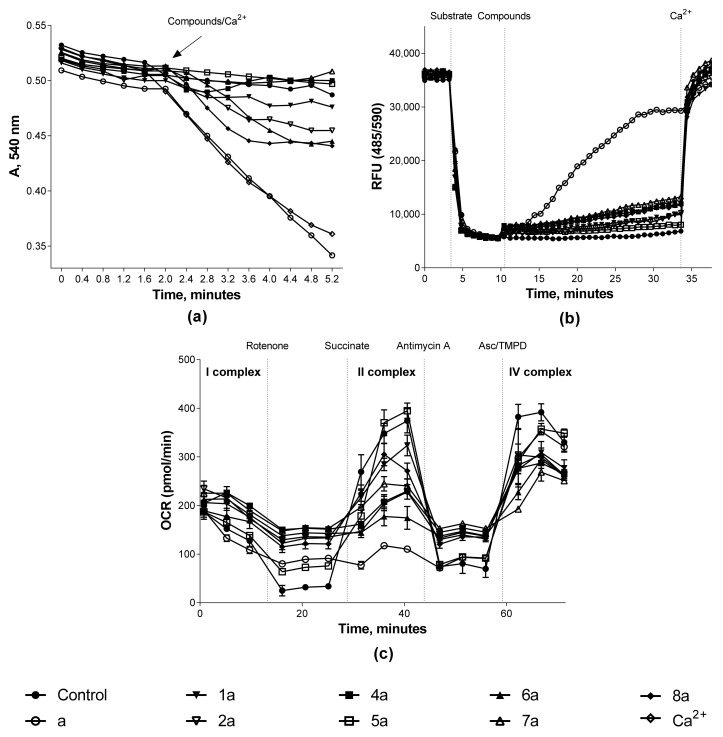
Effect of doxorubicin conjugates with sesquiterpene lactones on the functional characteristics of rat heart mitochondria. Kinetic curves of (**a**)—mitochondrial swelling and (**b**)—changes in the transmembrane potential of organelles (0.5 mg/mL) under the influence of the test compounds at 100 μM; the concentration of Ca^2+^ ions was 25 μM; (**c**)—changes in the OCR by organelles (10 μg/well) treated with the test compounds at 30 μM with the addition of modulators: rotenone (2 μM), potassium succinate (2 μM), antimycin A (4 μM), and ascorbate/TMPD (0.5 μM). Data for any experiment were obtained by averaging three repeated measurements for each compound.

The study of the effect of synthesized compounds on the mitochondria functional characteristics was carried out by analyzing three parameters: the measurement of mitochondrial swelling, which characterizes the process of permeability transition pore opening, the transmembrane potential, and the work of the respiratory chain complexes. The results obtained are presented in the form of summarized quantitative data in Table 2 for a comparative analysis of “structure–activity”. Figure 6 shows the kinetic curves of the studied processes for high-quality visualization.

**Figure 7:**
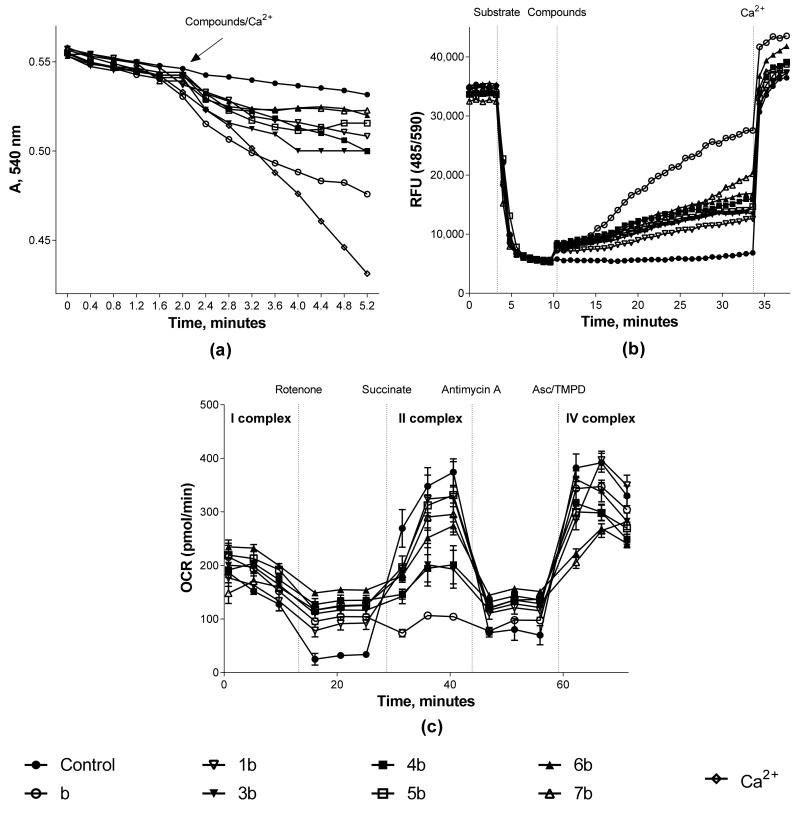
Effect of daunorubicin conjugates with sesquiterpene lactones on the functional characteristics of rat heart mitochondria. Kinetic curves of (**a**)—mitochondrial swelling and (**b**)—changes in the transmembrane potential of organelles (0.5 mg/mL) under the influence of the test compounds at 100 μM; the concentration of Ca^2+^ ions was 25 μM; (**c**)—changes in the OCR by organelles (10 μg/well) treated with the test compounds at 30 μM with the addition of modulators: rotenone (2 μM), potassium succinate (2 μM), antimycin A (4 μM), and ascorbate/TMPD (0.5 μM). Data for any experiment were obtained by averaging three repeated measurements for each compound.

As described earlier [10], doxo- and daunorubicin provoke the permeability transition pore opening and mitochondrial membrane depolarization. These data were confirmed by us in experiments on isolated rat heart mitochondria. When the initial anthracyclines were added to the isolated organelles, swelling was induced, which was comparable to the mitochondrial permeability pore formation under the action of a standard trigger of this process—calcium ions at a concentration of 25 μM (Figure 6A). Such a concentration allowed us to observe the maximum swelling rate in isolated rat heart mitochondria under our experimental conditions. However, no effect on mitochondrial swelling was observed for most conjugates (Table 2). The situation is similar to the effect on the mitochondria transmembrane potential. Doxo- and daunorubicin have a pronounced depolarizing effect, exceeding the action of conjugates in this process by more than three times (Table 2, Figure 6B and Figure 7B).

The functioning of the respiratory chain complexes of isolated rat heart mitochondria was studied using a cellular metabolism analyzer Agilent Seahorse XF96e Analyzer (Seahorse Bioscience, USA) in real time. The investigation of the oxygen consumption rate (OCR) was carried out under the influence of tissue respiration modulators.

It was found that both doxorubicin and daunorubicin had a pronounced inhibitory effect on complex II of the electron transport chain, which was expressed as a decrease in OCR by organelles by more than 70% (Table 2, Figure 6C andFigure 7C). A similar effect was recorded for doxorubicin with respect to the cytochrome-c-oxidase complex of the mitochondrial respiratory chain: OCR was reduced by 37.73 ± 0.34%. In turn, either no effect on the respiratory complex functioning or a decrease in OCR by mitochondria by no more than 40% was found for most of the synthesized conjugates. Only two compounds exhibited inhibitory activity, exceeding 50% (**6a** and **3b**) against respiratory chain complexes II and IV.

Thus, the results of studying the effect of the synthesized conjugates on the functional characteristics of rat heart mitochondria suggest that doxo- and daunorubicin modification with natural sesquiterpene lactones carried out by us may be a prerequisite for reducing the toxic effect on healthy cells, in particular, cardiomyocytes, against the background of similar cytotoxicity with the original anthracyclines.

As is known, cancerogenesis occurs in a hypoxic microenvironment, while a distinctive feature of neoplastic cells already at the earliest stages of the disease development is a high anaerobic glycolysis rate, characterized by intense glucose absorption followed by conversion to lactic acid in the absence of oxygen [33]. Thus, tumor cells receive the main part of the energy from this metabolic pathway, while no more than 10% of ATP is formed during glycolysis in physiologically healthy cells. This reprogrammed metabolism of neoplastic cells plays an important role in the development of malignant neoplasms, due to which this cell type has unlimited division ability, metastasis, and reduction of their death by avoiding apoptosis [34].

In our work, information about the glycolytic activity of the tumor cell culture HeLa under the action of the studied compounds was obtained by measuring the extracellular acidification rate during the sequential injection of metabolic modulators by the glycolysis stress test.

**Figure 8:**
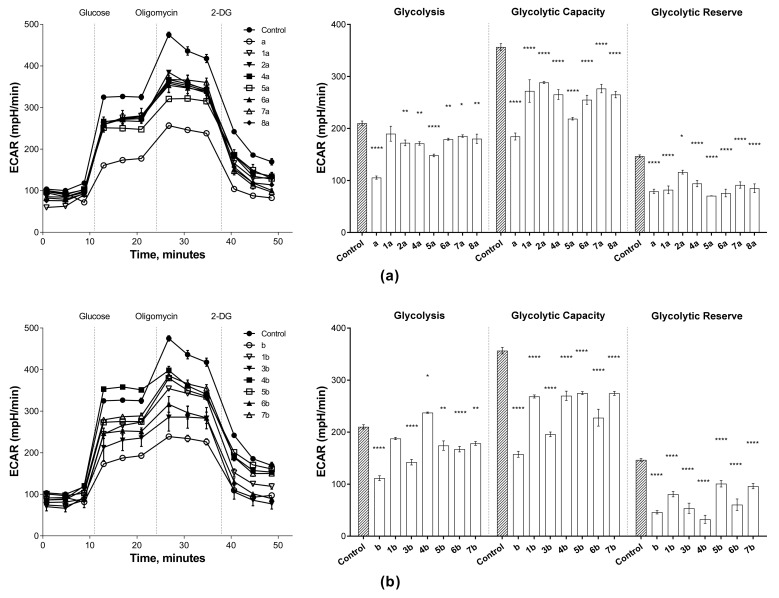
Effect of doxorubicin (**a**) and daunorubicin (**b**) conjugates with sesquiterpene lactones on the glycolytic profile of HeLa cells (density was 3 * 10^6^ cells/well). The concentration of the test substances was 30 μM, glucose—10 mM, oligomycin—1 μM, 2-fluoro-2-deoxy-D-glucose—25 mM. Data are presented as the extracellular acidification rate of the medium: as the mean ± SEM (three repeated measurements for each compound). Asterisks (*, **, ****) indicate a statistically significant (*p* < 0.05, *p* < 0.01, *p* < 0.0001) difference between the control and the studied compounds (one-way ANOVA, Dunnett’s test).

It was found that all studied substances inhibited the process of anaerobic glycolysis (Figure 8), which is expressed in their suppression of glycolytic function parameters such as glycolysis, glycolytic capacity, and glycolytic reserve. At the same time, the doxorubicin derivative with epoxy-alantolactone (**5a**) and the daunorubicin conjugate with alantolactone (**3b**) had the most pronounced ability to reduce the acidification rate of the medium by cells.

Thus, we were the first to obtain bipharmacophore molecules containing in their structure a fragment of the known anthracycline antibiotics—daunorubicin and doxorubicin—with natural sesquiterpene lactones. When studying the biological activity of the synthesized compounds, a reduced damaging effect on the rat heart mitochondria functioning was observed with equal and, in some cases, higher cytotoxicity and glycolysis inhibition by anthracycline antibiotic conjugates with sesquiterpene lactones in comparison with doxo- and daunorubicin. These results allow us to confirm the assumption that the chemical modification of the anthracycline antibiotic doxo- and daunorubicin molecules by sesquiterpene lactones can be a promising strategy for creating potential antitumor chemotherapeutic drugs with a pronounced cytotoxic effect on tumor cells and a reduced damaging effect on healthy cells of the human organism.

## Figures and Tables

**Table 1:** In vitro cytotoxicity daunorubicin and doxorubicin conjugates with sesquiterpene lactones.

Compound	IC_50_, µM				
	A549	HCT116	MCF7	RD	HEK293
**1**	32.04 ± 3.24	11.31 ± 0.27	17.51 ± 0.60	10.37 ± 0.79	74.03 ± 0.51
**2**	36.73 ± 1.43	10.57 ± 0.04	13.15 ± 0.93	5.48 ± 0.20	36.47 ± 0.07
**3**	23.12 ± 1.18	34.52 ± 3.51	17.92 ± 0.68	8.82 ± 0.14	35.87 ± 0.48
**4**	83.51 ± 0.26	21.40 ± 0.32	31.87 ± 0.14	18.60 ± 0.21	105.68 ± 4.13
**5**	21.49 ± 0.75	5.12 ± 0.05	11.41 ± 0.32	4.81 ± 0.12	38.10 ± 1.08
**6**	50.28 ± 1.11	9.75 ± 0.57	24.03 ± 0.80	8.77 ± 0.01	18.47 ± 0.20
**6**	61.71 ± 2.40	10.80 ± 0.18	38.55 ± 0.86	16.78 ± 0.28	29.77 ± 0.05
**8**	59.86 ± 2.26	4.99 ± 0.06	4.31 ± 0.02	4.94 ± 0.09	9.07 ± 0.25
**a**	0.38 ± 0.02	0.14 ± 0.01	0.46 ± 0.03	0.29 ± 0.02	6.78 ± 0.76
**1a**	2.21 ± 0.06	2.81 ± 0.05	11.11 ± 0.17	2.41 ± 0.03	19.82 ± 0.42
**2a**	3.29 ± 0.11	4.55 ± 0.03	26.76 ± 0.65	2.67 ± 0.11	29.83 ± 0.18
**4a**	1.86 ± 0.12	1.07 ± 0.01	5.65 ± 0.22	2.76 ± 0.02	11.73 ± 0.10
**5a**	4.52 ± 0.57	2.52 ± 0.02	3.03 ± 0.07	2.47 ± 0.07	4.50 ± 0.32
**6a**	0.88 ± 0.11	0.25 ± 0.04	2.94 ± 0.02	1.18 ± 0.01	0.98 ± 0.23
**7a**	5.43 ± 0.16	0.92 ± 0.00	6.03 ± 0.11	4.22 ± 0.10	10.49 ± 0.88
**8a**	1.87 ± 0.07	0.43 ± 0.02	2.34 ± 0.01	1.03 ± 0.02	16.42 ± 1.07
**b**	0.33 ± 0.01	0.12 ± 0.00	0.84 ± 0.17	0.63 ± 0.03	12.31 ± 1.17
**1b**	0.93 ± 0.06	0.28 ± 0.00	3.95 ± 0.03	0.96 ± 0.02	3.43 ± 0.04
**3b**	1.42 ± 0.05	1.01 ± 0.01	1.24 ± 0.23	0.59 ± 0.05	6.37 ± 0.24
**4b**	0.27 ± 0.01	0.02 ± 0.00	1.99 ± 0.27	0.63 ± 0.02	11.41 ± 0.53
**5b**	1.19 ± 0.02	1.30 ± 0.02	1.84 ± 0.06	0.80 ± 0.00	18.19 ± 0.18
**6b**	0.56 ± 0.01	0.26 ± 0.01	1.47 ± 0.04	0.41 ± 0.02	3.68 ± 0.16
**7b**	2.01 ± 0.09	0.20 ± 0.01	1.90 ± 0.07	1.95 ± 0.01	6.42 ± 0.21

Values were obtained by averaging three repeated measurements for each concentration.

**Table 2:** Effect of daunorubicin and doxorubicin conjugates with sesquiterpene lactones on the functional characteristics of rat heart mitochondria.

Compound	Work of Respiratory Chain Complexes, % Decrease in OCR of Control		Mitochondrial Swelling, % of Ca^2+^ -Induced Swelling	Mitochondrial Membrane Depolarization, % of Control
	II	IV		
**a**	70.54 ± 0.64	37.73 ± 0.34	116.20 ± 10.98	61.55 ± 4.49
**1a**	27.61 ± 5.71	24.52 ± 7.61	—	—
**2a**	39.11 ± 4.05	20.45 ± 5.85	31.51 ± 8.35	—
**4a**	38.58 ± 1.05	27.69 ± 2.93	—	14.04 ± 1.48
**5a**	—	23.99 ± 4.41	—	—
**6a**	53.34 ± 6.31	40.50 ± 4.05	54.22 ± 5.49	15.39 ± 0.42
**7a**	33.21 ± 3.84	38.16 ± 0.84	—	17.72 ± 0.69
**8a**	27.58 ± 4.30	25.95 ± 4.43	47.84 ± 4.99	13.34 ± 1.72
**b**	72.12 ± 0.96	—	40.98 ± 3.86	56.81 ± 3.19
**1b**	—	—	—	15.77 ± 0.20
**3b**	57.77 ± 1.75	—	26.45 ± 7.14	17.99 ± 0.57
**4b**	46.22 ± 9.50	—	27.75 ± 4.25	24.82 ± 2.52
**5b**	—	21.56 ± 3.66	—	21.55 ± 1.04
**6b**	26.74 ± 4.56	41.69 ± 2.13	—	27.55 ± 1.54
**7b**	21.04 ± 4.22	46.08 ± 3.42	—	36.76 ± 2.90

Data in the table are presented as mean ± SEM (three repeated measurements for each compound in any experiment). “—” indicates activity absence for compounds (≤10%) in a particular test.

## Data Availability

The following are available online at Supplementary Materials containing NMR spectra for compounds.

## Supplementary Materials

The following are available online at https://www.mdpi.com/article/10.3390/biomedicines9050547/s1.
