# Supplementary material for: N-Alkylation of Anthracycline Antibiotics by Natural Sesquiterpene Lactones as a Way to Obtain Antitumor Agents with Reduced Side Effects
Source: Biomedicines. 2021 May 13;9(5):547. doi: 10.3390/biomedicines9050547 (PMC8153121; doi:10.3390/biomedicines9050547)
Supplement: Supplementary file 1 [file biomedicines-09-00547-s001.zip › biomedicines-1169422-supplementary.pdf]

N-alkylation of anthracycline antibiotics by natural sesquiterpene lactones as a way to obtain antitumor agents with reduced side effects

Margarita Neganova [1], Alexey Semakov [1], Yulia Aleksandrova [1], Ekaterina Yandulova [1], Sergey Pukhov [1], Lada Anikina [1] and Sergey Klochkov [1]*

[1] Institute of Physiologically Active Compounds of Russian Academy of Sciences, 142432, Chernogolovka, Russia; klochkov@ipac.ac.ru (S.K.); l_vok@list.ru (A.S.); yulia.aleks.97@mail.ru (Yu.A.); yandulovacaterina@gmail.com (E.Ya.); pukhov.sergey@gmail.com (S.P.); neganova83@mail.ru (M.N.)

* Correspondence: klochkov@ipac.ac.ru; Tel.: +7-(496)5242525

^{1}H and ^{13}C NMR spectra were recorded on Bruker AVANCE III instruments (operating frequency 500.13 and 125.78 MHz) in CDCl3, internal standard is the residual solvent signal, subscripts “alpha” and “beta” denote nonequivalent protons at one carbon atom. To refine the multiplicity in the proton spectra analysis, we used the multiplication by the TRAF function. For some weakly intense ^{13}C spectra, the Convolution difference was used to increase the signal-to-noise ratio.

18.03.2021 20:38:02

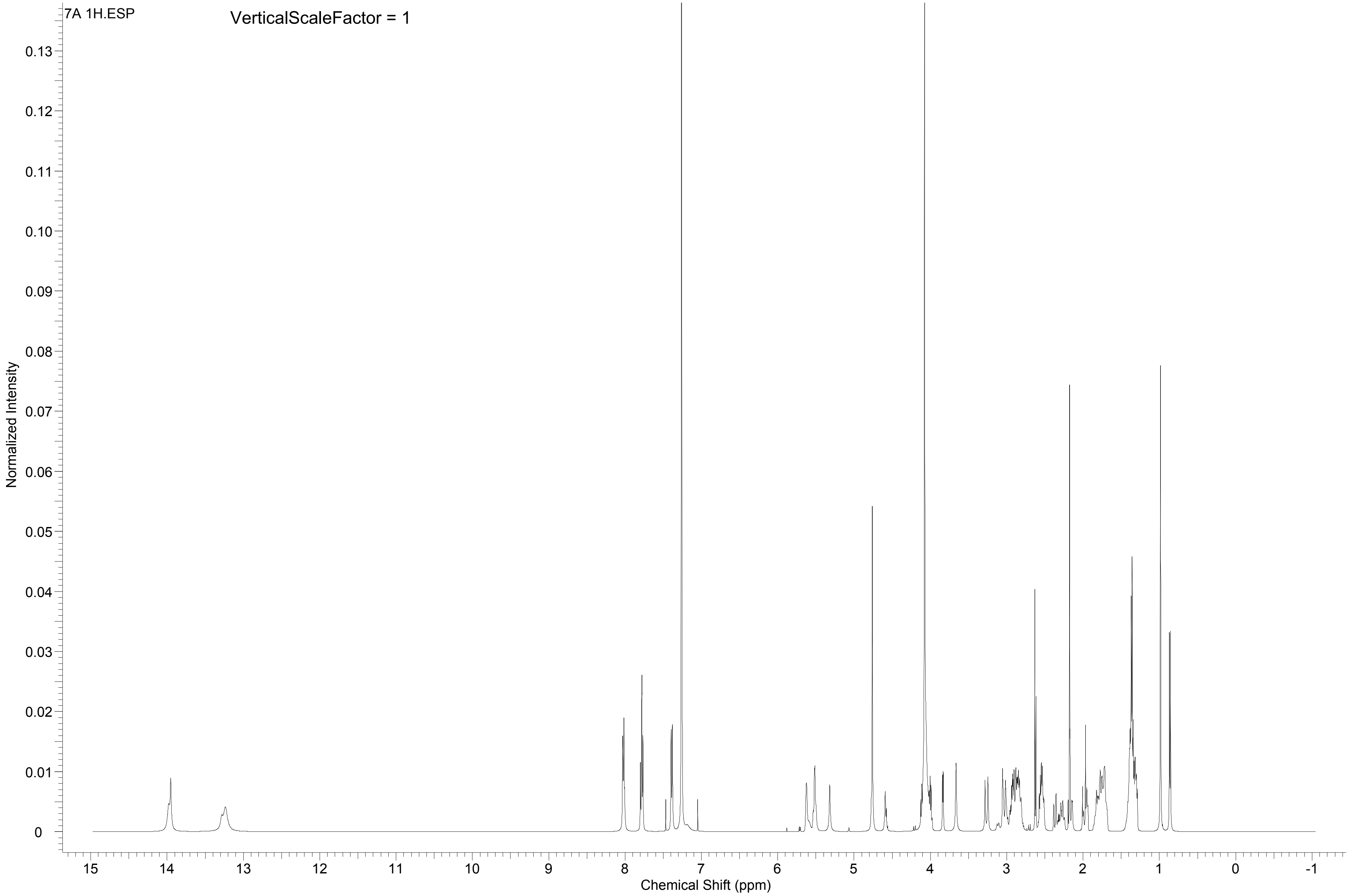

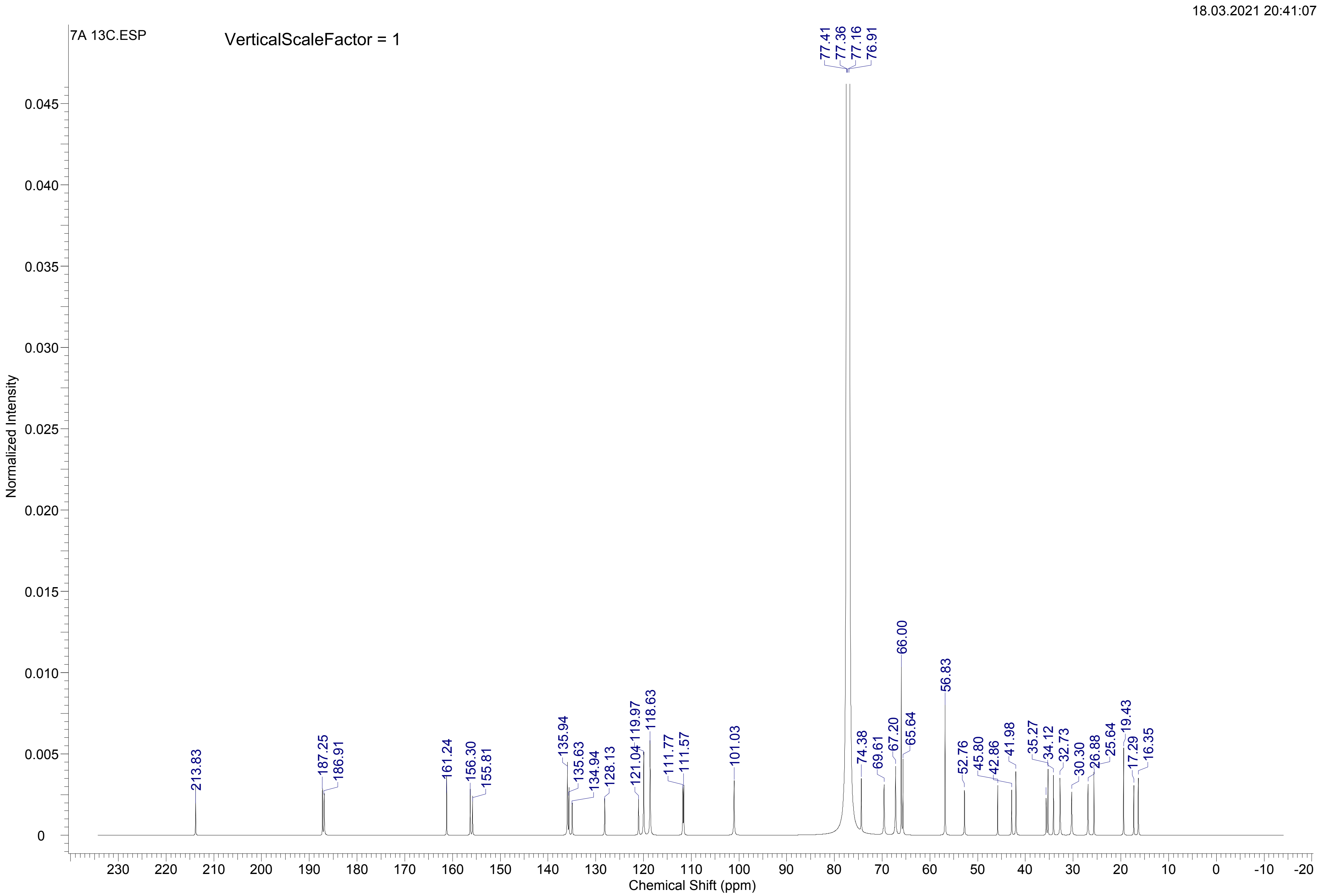
18.03.2021 20:41:07
7A 13C.ESP
VerticalScaleFactor = 1
77.41
77.36
77.16
76.91
66.00
56.83
213.83
187.25
186.91
161.24
156.30
155.81
135.94
135.63
134.94
128.13
121.04
119.97
118.63
111.77
111.57
101.03
74.38
69.61
67.20
65.64
52.76
45.80
42.86
41.98
35.27
34.12
32.73
30.30
26.88
25.64
19.43
17.29
16.35
Normalized Intensity
0.045
0.040
0.035
0.030
0.025
0.020
0.015
0.010
0.005
0
230 220 210 200 190 180 170 160 150 140 130 120 110 100 90 80 70 60 50 40 30 20 10 0 -10 -20
Chemical Shift (ppm)

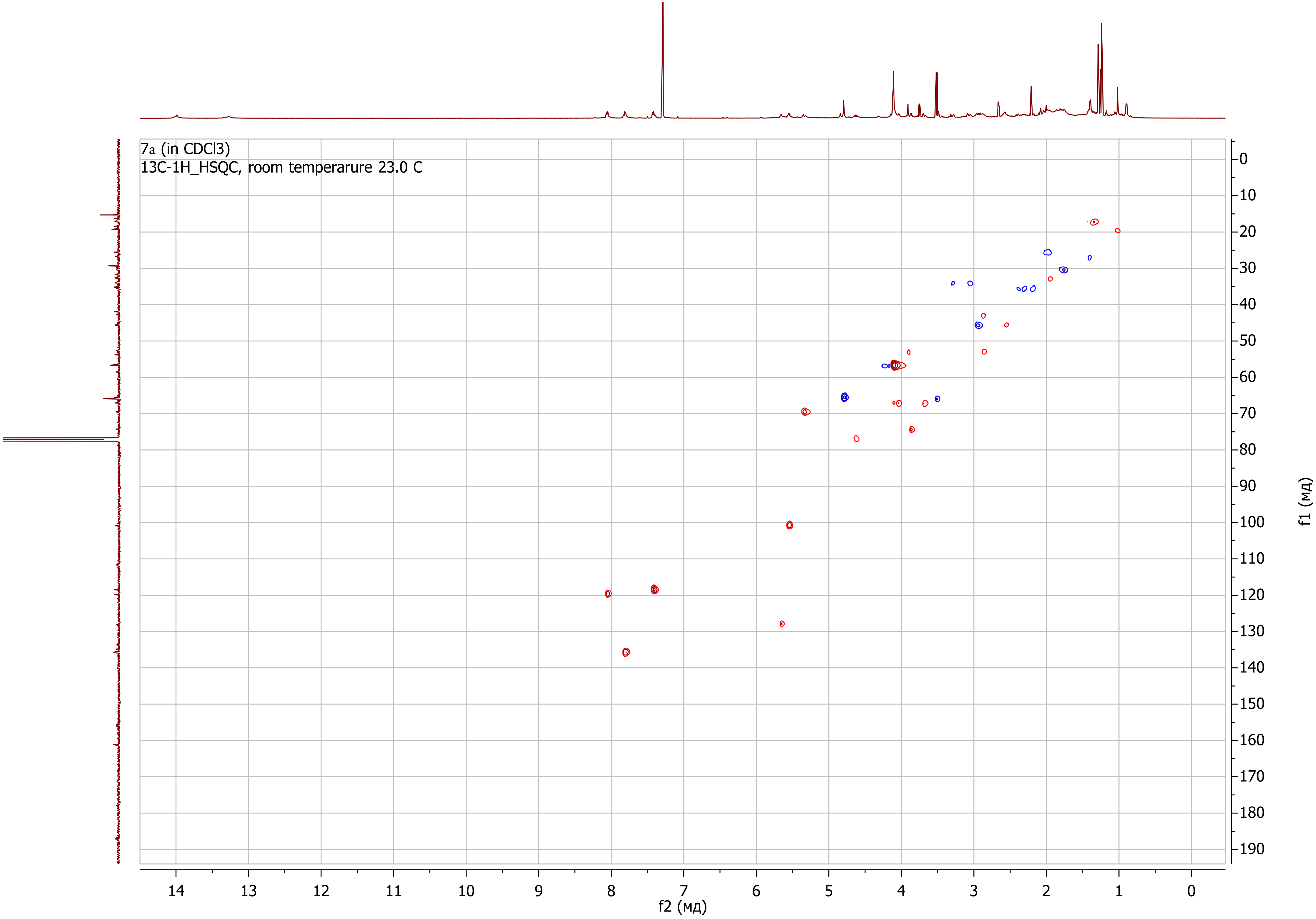

7a (in CDCl3)
13C-1H_HSQC, room temperarure 23.0 C
f2 (мд)
f1 (мд)

18.03.2021 20:41:54

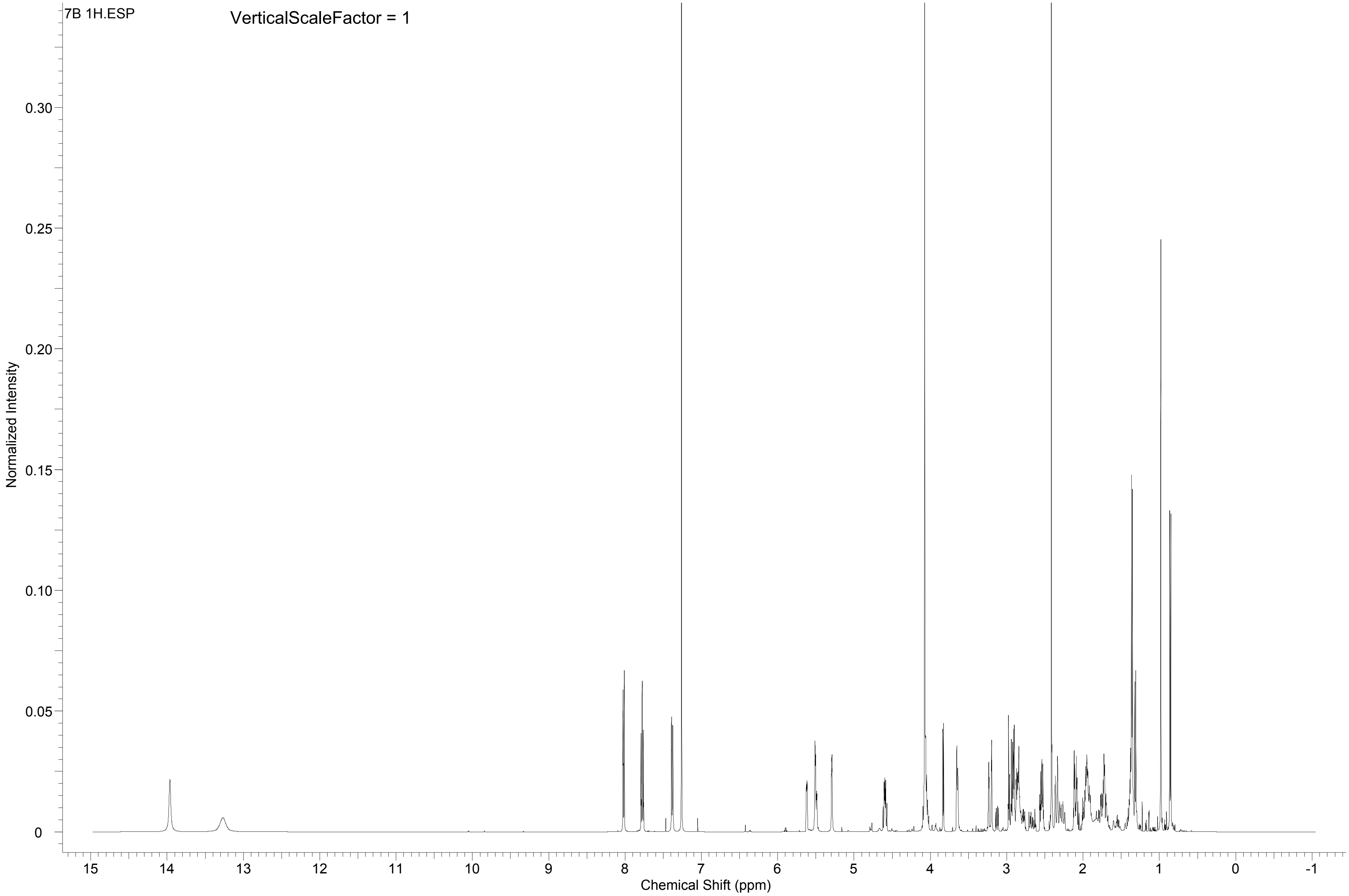

18.03.2021 20:45:42

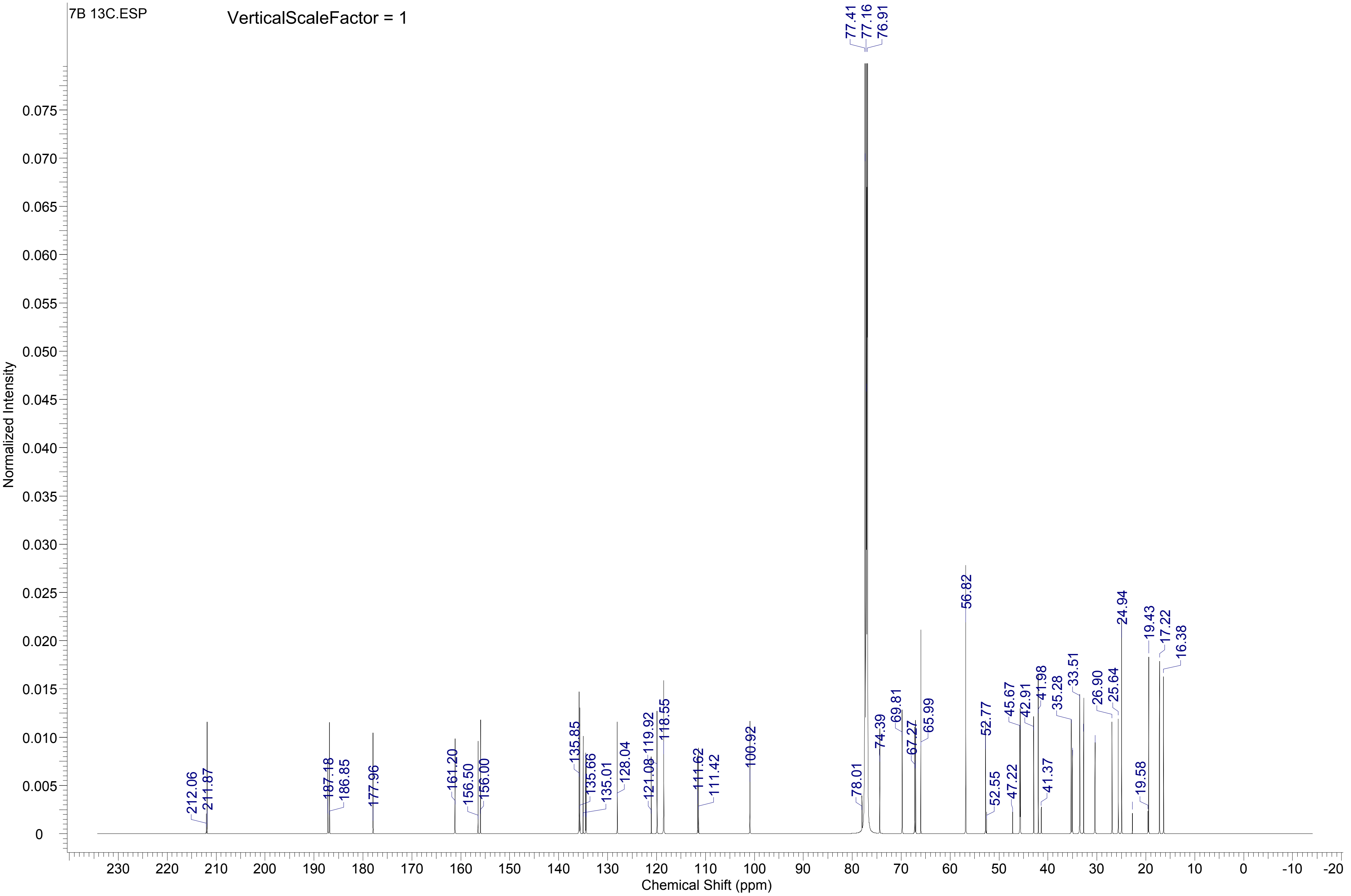

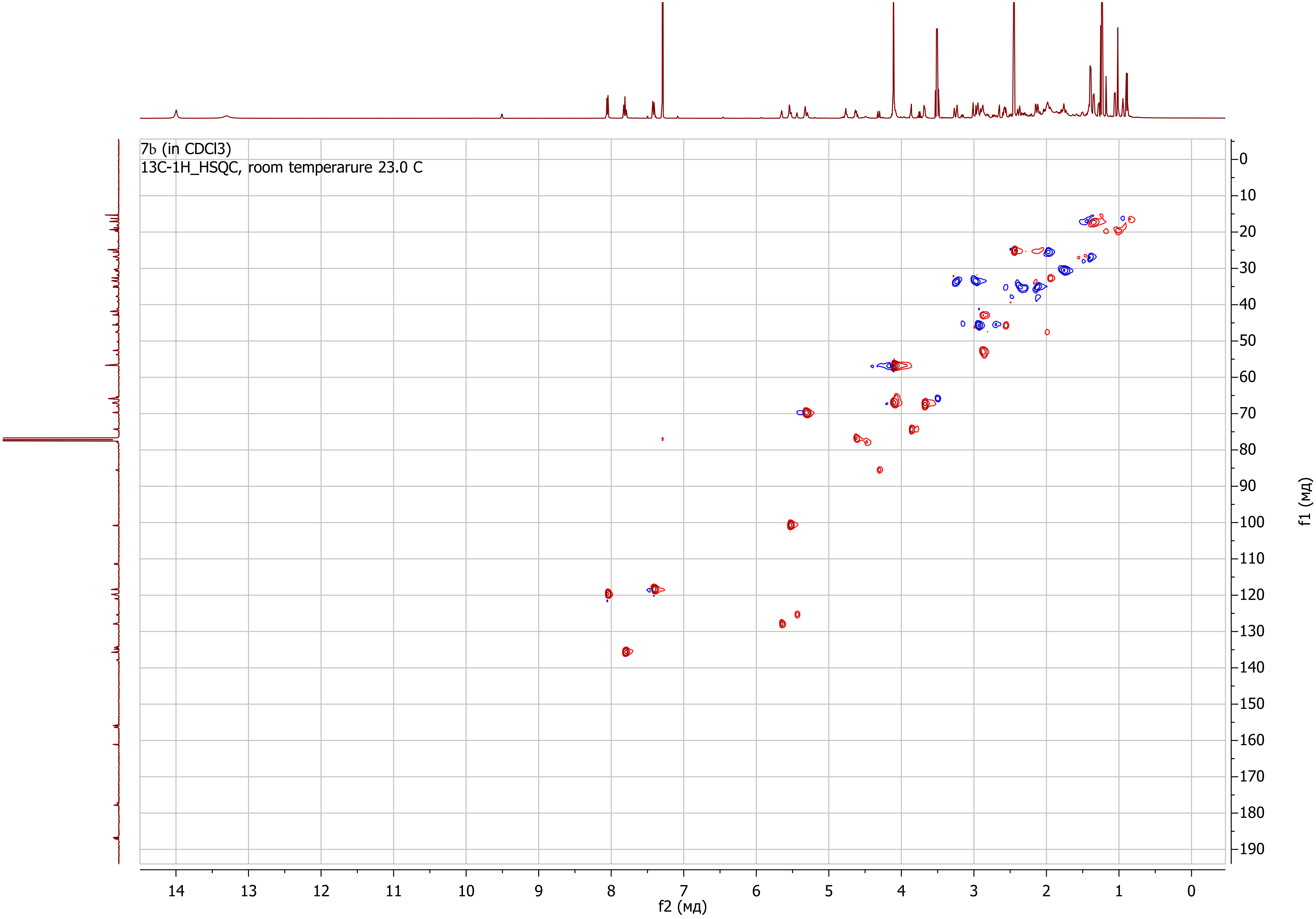
7b (in CDCl3)
13C-1H_HSQC, room temperarure 23.0 C
f2 (мд)
f1 (мд)

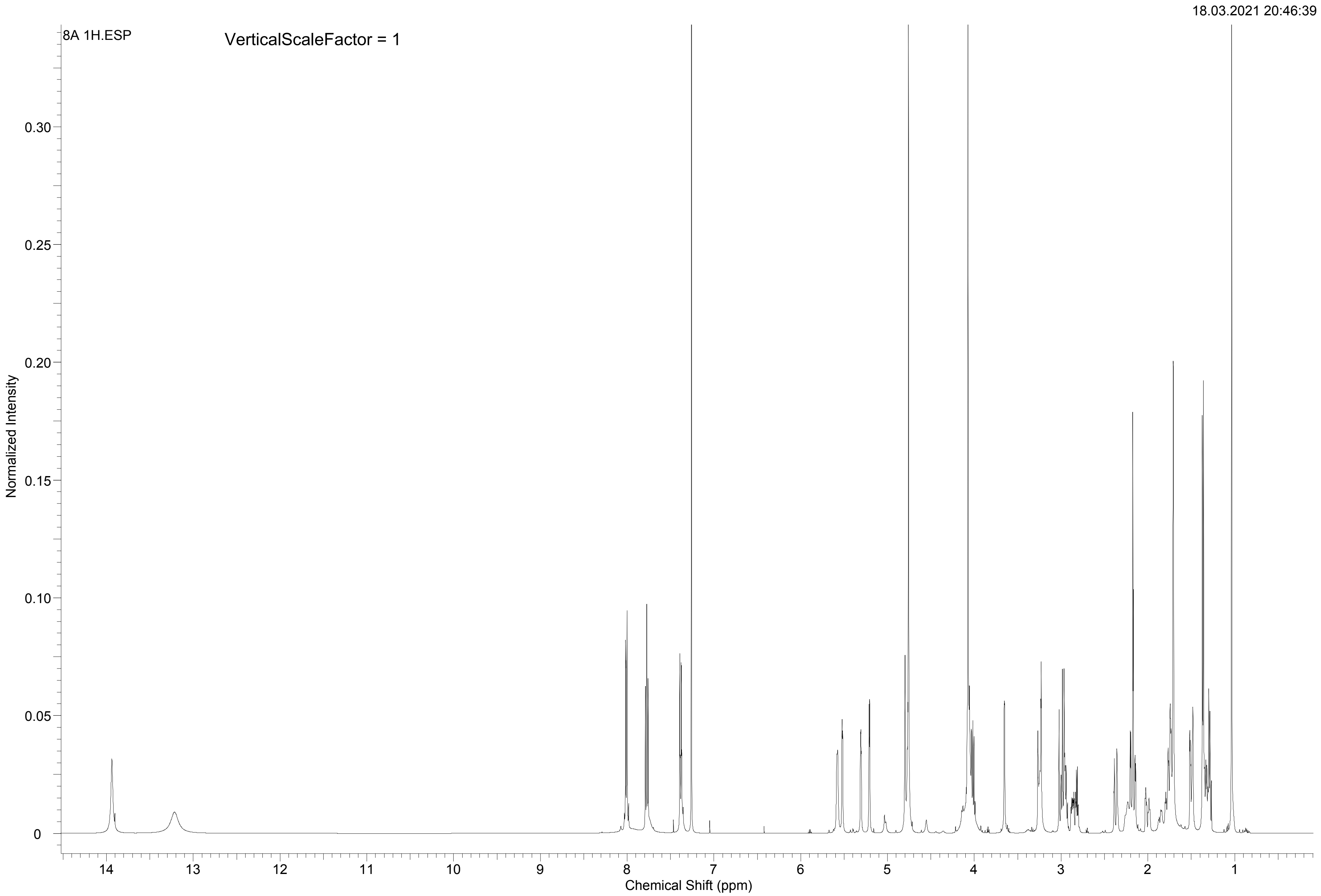
18.03.2021 20:46:39
8A 1H.ESP
VerticalScaleFactor = 1
Normalized Intensity
0.30
0.25
0.20
0.15
0.10
0.05
0
14
13
12
11
10
9
8
7
6
5
4
3
2
1
Chemical Shift (ppm)

18.03.2021 20:47:34

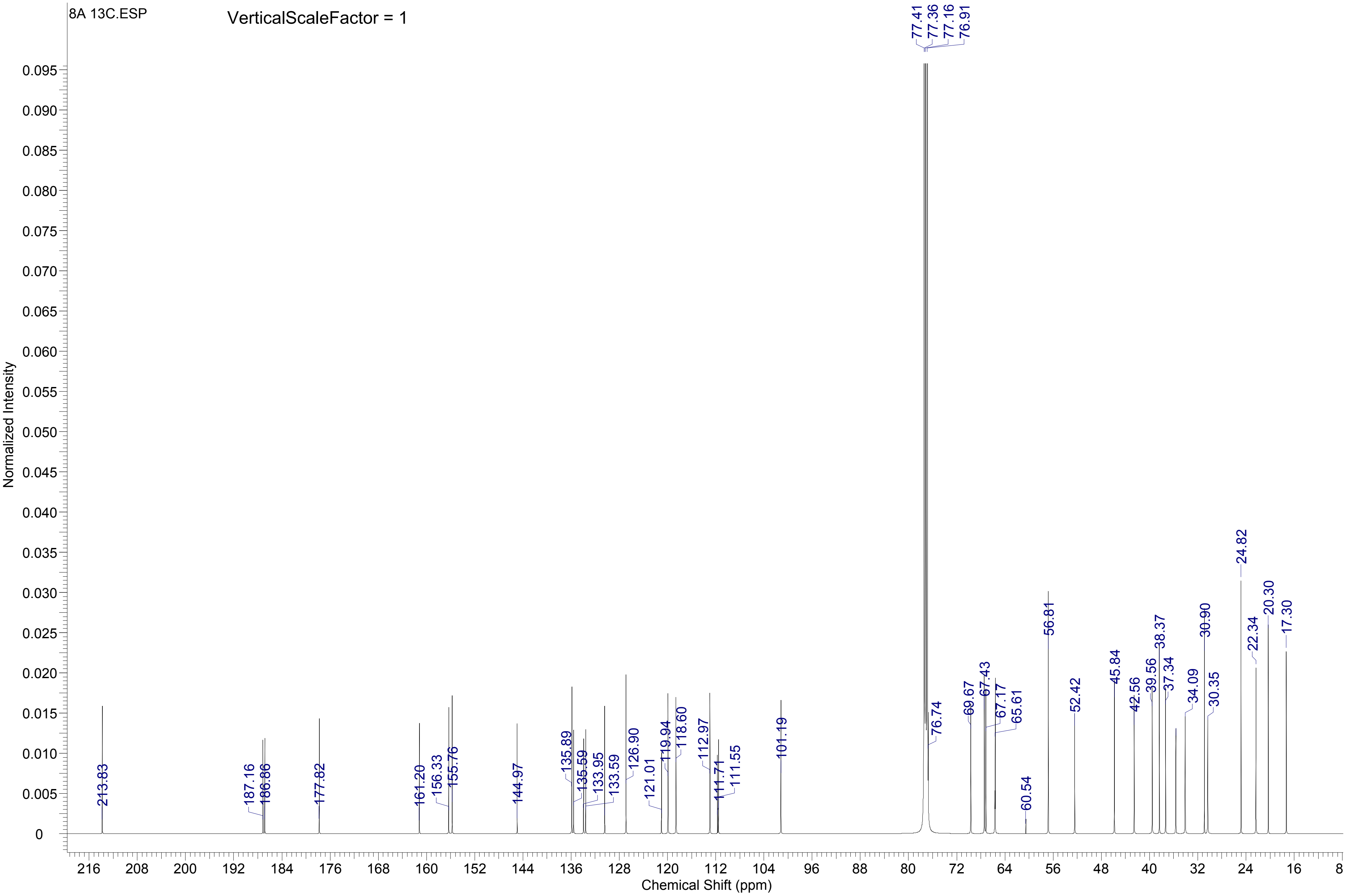

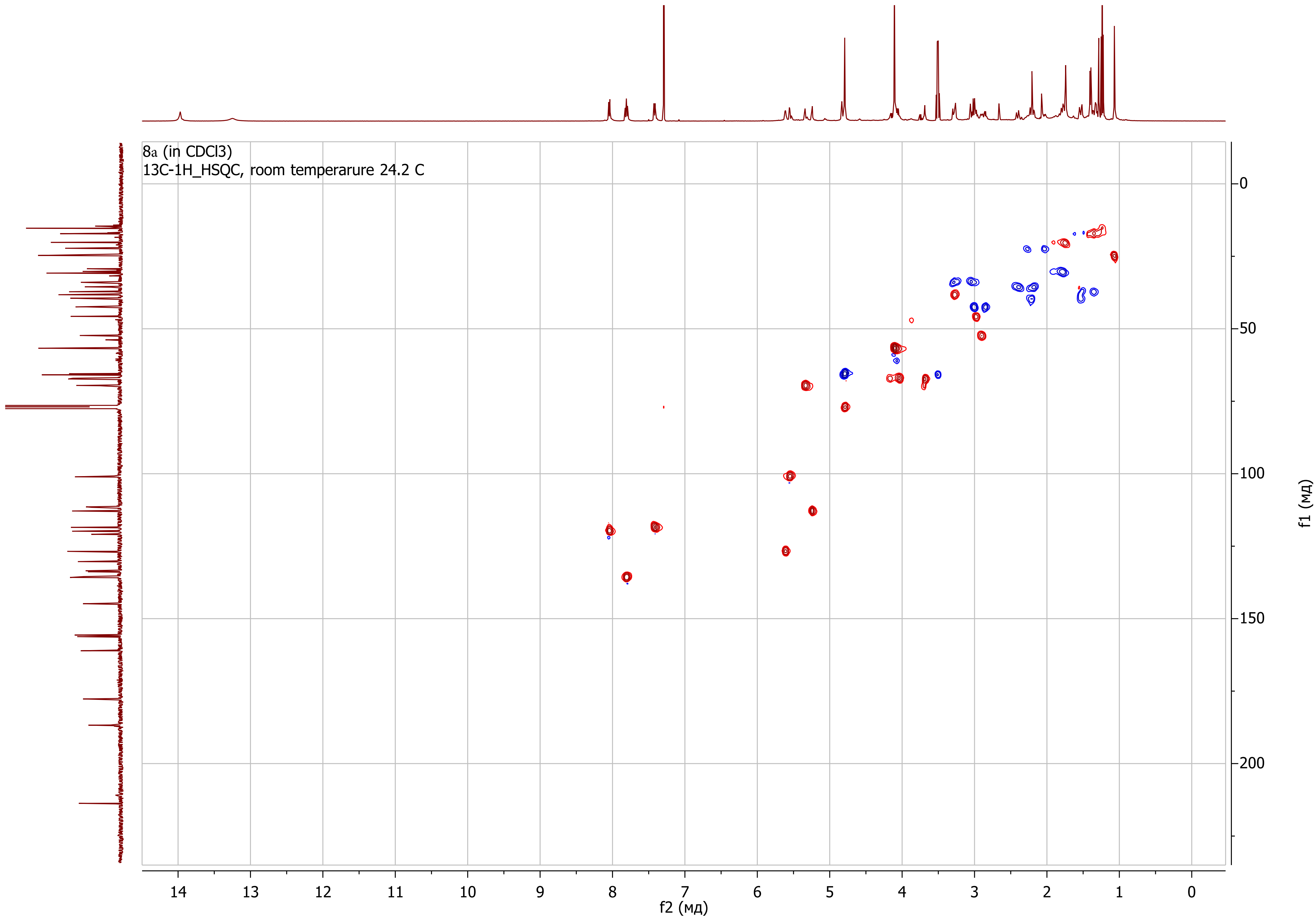
8a (in CDCl3)
13C-1H_HSQC, room temperarure 24.2 C
f2 (мд)
f1 (мд)
14
13
12
11
10
9
8
7
6
5
4
3
2
1
0
50
100
150
200

18.03.2021 20:49:13

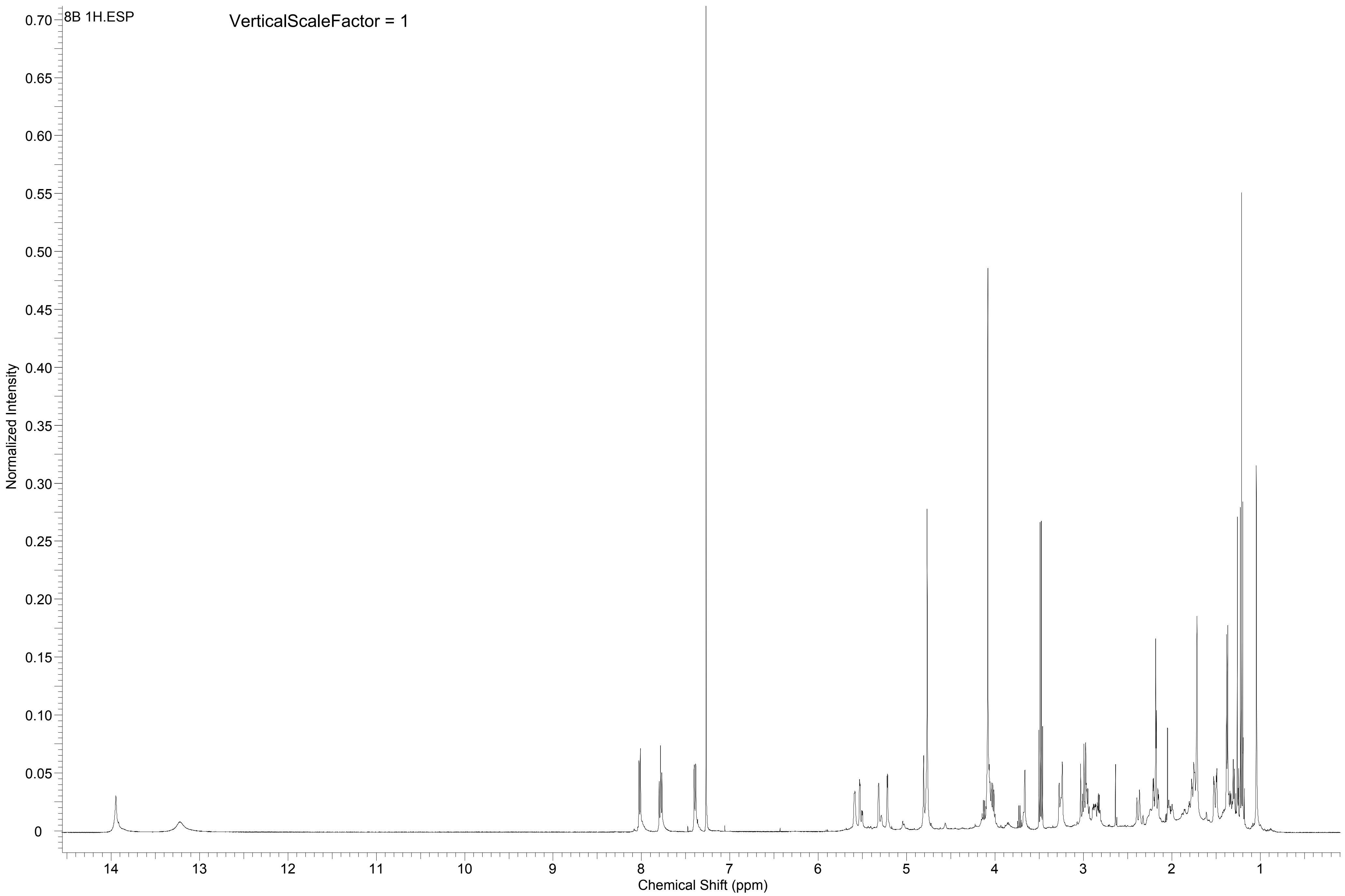

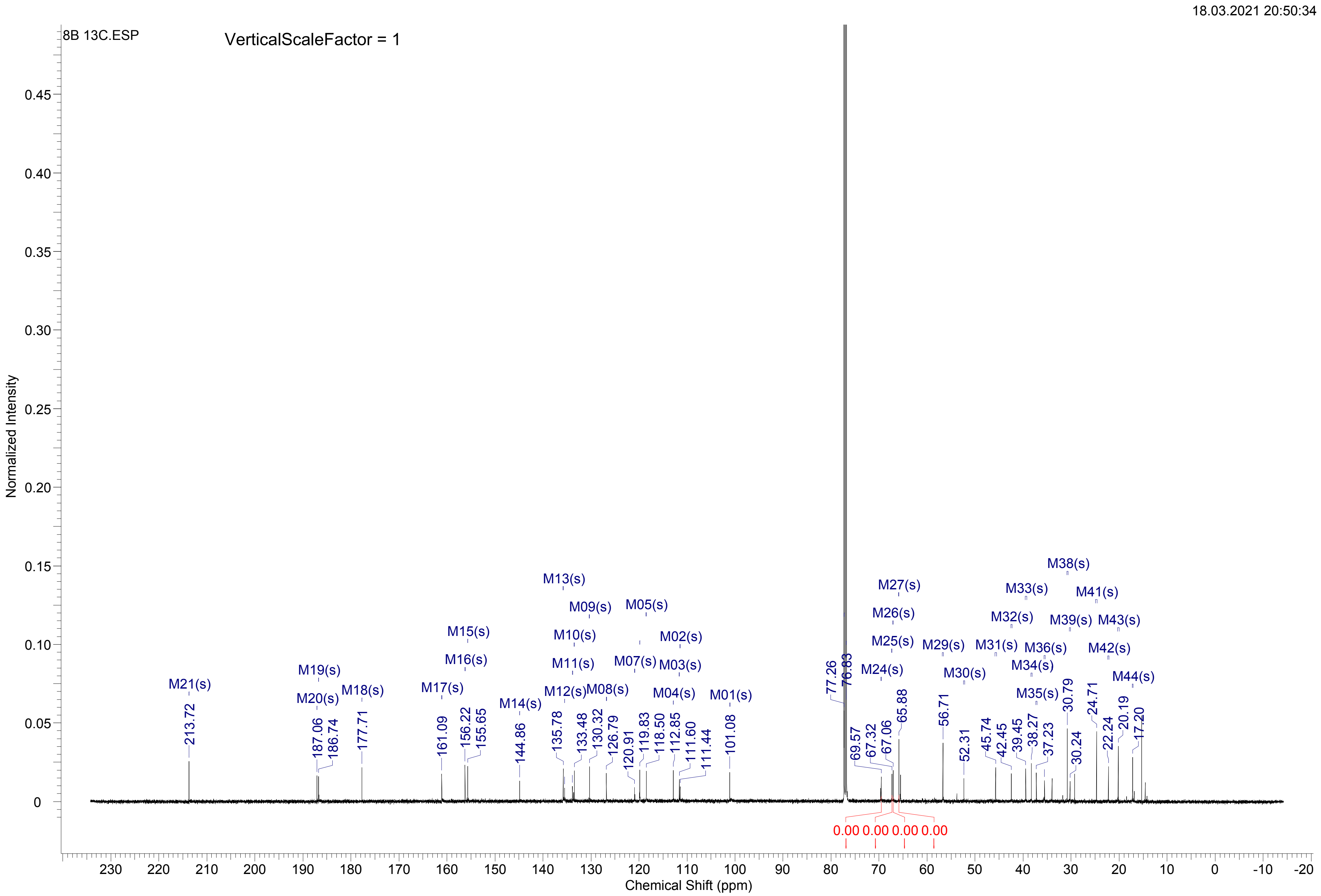

18.03.2021 20:50:34
8B 13C.ESP
VerticalScaleFactor = 1
Normalized Intensity
Chemical Shift (ppm)
213.72
187.06
186.74
177.71
161.09
156.22
155.65
144.86
135.78
133.48
130.32
126.79
120.91
119.83
118.50
112.85
111.60
111.44
101.08
77.26
76.83
69.57
67.32
67.06
65.88
56.71
52.31
45.74
42.45
39.45
38.27
37.23
30.79
30.24
24.71
22.24
20.19
17.20
0.00 0.00 0.00 0.00

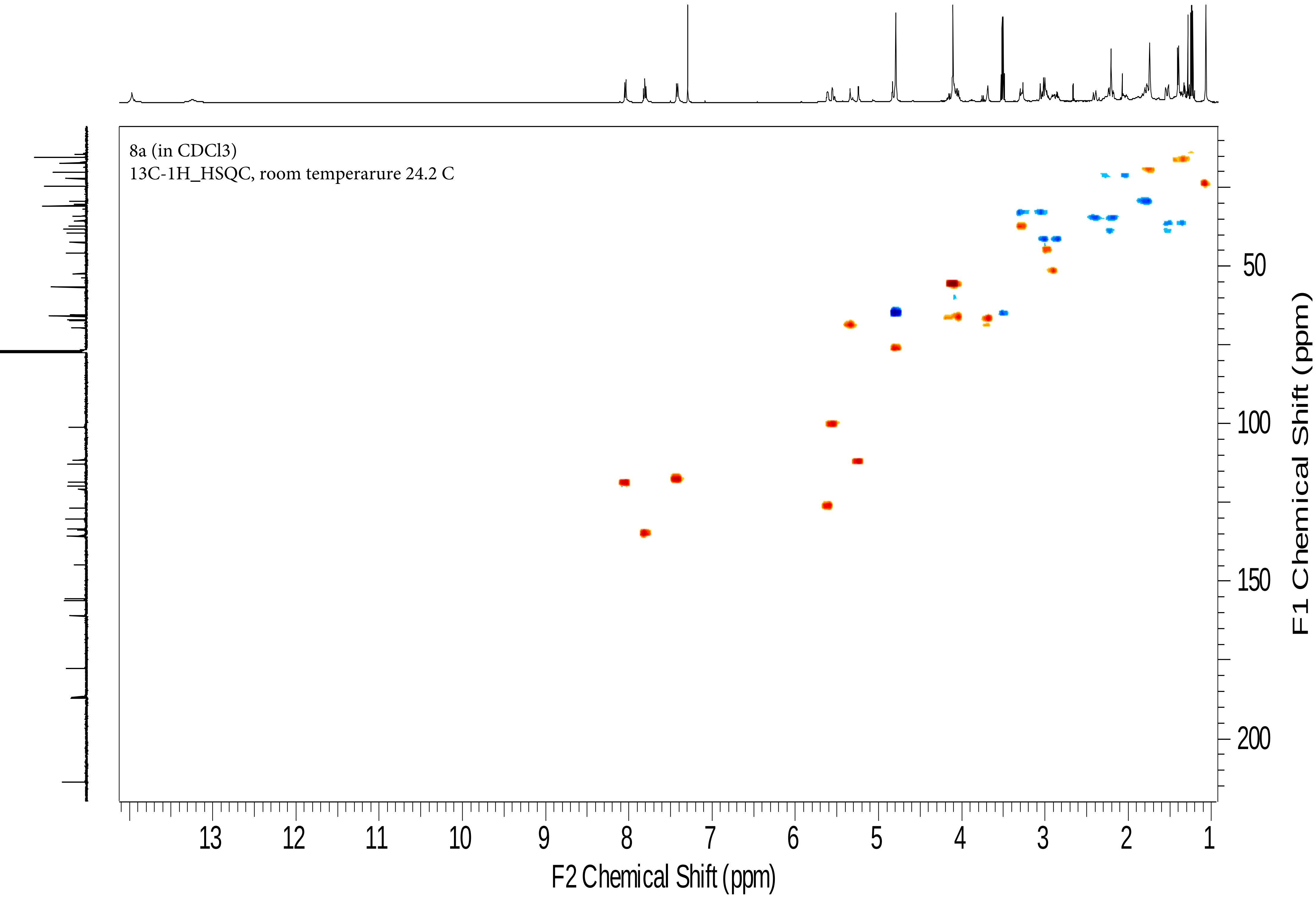

8a (in CDCl3)
13C-1H_HSQC, room temperarure 24.2 C
F1 Chemical Shift (ppm)
50
100
150
200
13
12
11
10
9
8
7
6
5
4
3
2
1
F2 Chemical Shift (ppm)
